# PCSK9 deficiency reduces atherosclerosis, apolipoprotein B secretion, and endothelial dysfunction[S]

**DOI:** 10.1194/jlr.M078360

**Published:** 2017-11-27

**Authors:** Hua Sun, Ronald M. Krauss, Jeffrey T. Chang, Ba-Bie Teng

**Affiliations:** Research Center for Human Genetics, Brown Foundation Institute of Molecular Medicine* University of Texas Health Science Center at Houston, Houston, TX; Department of Integrative Biology and Pharmacology,§ University of Texas Health Science Center at Houston, Houston, TX; Children’s Hospital Oakland Research Institute, † Oakland, CA; University of Texas MD Anderson Cancer Center,** University of Texas Health Science Center at Houston Graduate School of Biomedical Sciences, Houston, TX

**Keywords:** proprotein convertase subtilisin/kexin type 9, autophagy, low density lipoprotein

## Abstract

Proprotein convertase subtilisin/kexin type 9 (PCSK9) interacts directly with cytoplasmic apoB and prevents its degradation via the autophagosome/lysosome pathway. This process affects VLDL and LDL production and influences atherogenesis. Here, we investigated the molecular machinery by which PCSK9 modulates autophagy and affects atherogenesis. We backcrossed *Pcsk9*^−/−^ mice with atherosclerosis-prone *Ldlr*^−/−^*Apobec1*^−/−^ (LDb) mice to generate *Ldlr*^−/−^*Apobec1*^−/−^*Pcsk9*^−/−^ (LTp) mice. Deletion of PCSK9 resulted in decreased hepatic apoB secretion, increased autophagic flux, and decreased plasma levels of IDL and LDL particles. The LDLs from LTp mice (LTp-LDLs) were less atherogenic and contained less cholesteryl ester and phospholipids than LDb-LDLs. Moreover LTp-LDLs induced lower endothelial expression of the genes encoding *TLR2*, *Lox-1*, *ICAM-1*, *CCL2*, *CCL7*, *IL-6*, *IL-1β*, *Beclin-1*, *p62*, and *TRAF6*. Collectively, these effects were associated with substantially less atherosclerosis development (>4-fold) in LTp mice. The absence of PCSK9 in LDb mice results in decreased lipid and apoB levels, fewer atherogenic LDLs, and marked reduction of atherosclerosis. The effect on atherogenesis may be mediated in part by the effects of modified LDLs on endothelial cell receptors and proinflammatory and autophagy molecules. These findings suggest that there may be clinical benefits of PCSK9 inhibition due to mechanisms unrelated to increased LDL receptor activity.

Proprotein convertase subtilisin/kexin type 9 (PCSK9) binds to the LDL receptor (LDLR) and thereby promotes its intracellular degradation. This results in reduced hepatic LDL uptake and increased plasma LDL concentrations (1). In 2003, Abifadel et al. (2) described gain-of-function mutations in the PCSK9 genes, which cause severe hypercholesterolemia. Studies in families from Utah (3), Norway (4), and Britain (5) confirmed these findings. Loss-of-function mutations in PCSK9 result in a reduction in LDL cholesterol (6) and are associated with protection from the development of coronary heart disease (7). Thus, the stage was set for the development of therapies that could inhibit PCSK9, lower LDL cholesterol, and consequently reduce atherothrombotic events, as was recently shown for one such therapeutic agent, the humanized PCSK9 monoclonal antibody, evolocumab (8).

Denis et al. (9) have shown that PCSK9 contributes to the development of atherosclerosis in mice in an LDLR-dependent manner. Ason et al. (10) have added that both LDLR and apoE are required to mediate the PCSK9 effect on atherosclerosis in mice. Thus, these two animal studies suggest that PCSK9 contributes to atherosclerosis development via an LDLR-dependent mechanism.

However, the regulation of LDLR by PCSK9 and the contributions of PCSK9 to atherogenesis are complex and not completely understood. Our laboratory has provided evidence that PCSK9 regulates apoB synthesis and secretion through an autophagic process and that this effect is independent of the LDLR (11). In addition, Tavori et al. (12) have shown that PCSK9 increases hepatic apoB-containing lipoproteins in an LDLR-independent fashion. Levy et al. (13) have demonstrated that PCSK9 regulates apoB48 secretion and cholesterol metabolism in CaCo-2 cells independently of the LDLR. PCSK9 also stimulates intestinal microsomal triglyceride (TG) transfer protein independently of the LDLR (14). Further, evolocumab has been shown to significantly reduce LDL cholesterol and apoB in patients with homozygous familial hypercholesterolemia with either one or two defective *LDLR* alleles or one defective and one null *LDLR* allele, although this effect was not seen in patients with homozygous null mutations (15). Taken together, these findings suggest that the effect of PCSK9 on LDL and apoB, and perhaps atherogenesis, are not entirely determined by the interaction of PCSK9 with the LDLR.

To define the effects of PCSK9 on the regulation of plasma apoB and atherogenesis more precisely, and to determine to what extent these effects may be LDLR dependent, the Pcsk9 gene was deleted from our atherosclerosis-prone mouse model, *Ldlr*^−/−^*Apobec1*^−/−^ (LDb) (11, 16–19), which lacks both LDLR and Apobec1, to produce a *Ldlr*^−/−^*Apobec1*^−/−^*Pcsk9*^−/−^ (LTp) triple KO mouse. In this work, we show that the absence of PCSK9 in LDb mice results in decreased production of hepatic apoB100-containing lipoproteins with modified lipid composition in conjunction with decreased atherogenesis via LDLR-independent mechanisms. We also show that deficiency of PCSK9 regulates apoB metabolism by modulating the autophagy signaling pathway and increasing autophagy flux in the liver.

## MATERIALS AND METHODS

### Animal studies

C57BL/6J, *Ldlr*^−/−^, and *Pcsk9*^−/−^ mice were purchased from Jackson Laboratory (Bar Harbor, ME). The *Pcsk9*^−/−^ mice were backcrossed with C57BL/6J to eight generations. The *Ldlr*^−/−^ mice were backcrossed with C57BL/6J to 10 generations. The LDb mice were generated as described previously (11, 16–20). The triple KO LTp mice were generated by crossing *Pcsk9*^−/−^ mice with LDb mice, with confirmation of the genotype by PCR. Mice were kept in a barrier facility with a 12:12 h dark-light cycle, and maintained on a standard laboratory chow diet. As male mice develop atherosclerosis earlier and more extensively compared with females (16, 19), only male mice were used for this study. The mice were bred to the required number at our strict barrier facility. All animal experiments were conducted in accordance with the guidelines of the Animal Protocol Review Committee of the University of Texas Health Science Center at Houston.

### Analysis of plasma lipid, plasma apoB, and fast protein LC lipoprotein levels

Plasma total cholesterol (TC), TGs, and phospholipids (PLs) were determined using the Cholesterol E, L-type Triglyceride M, and Phospholipid C kits (Wako Chemicals, Richmond, VA), respectively. Free cholesterol (FC) and cholesteryl ester (CE) were measured using an assay kit from Abcam (#Ab65359; Abcam, Cambridge, MA). Plasma apoB was separated using 6% proSieve-50 gel electrophoresis (FMC BioProducts, Rockland, ME). The respective protein was detected by anti-mouse apoB (Abcam; #Ab20737 at 1:3,000 dilution) Western blot analysis. The intensity of protein bands was semi-quantified using an Odyssey infrared imaging system (Li-COR, Lincoln, NE).

Pooled plasma (250 μl), usually obtained from an equal volume (50 μl) of five mice, was separated by fast protein LC (FPLC) on two Superose 6 columns connected in series (Thermo Fisher Scientific, Waltham, MA) (21, 22). Fractions (0.5 ml each) were eluted using PBS buffer at pH 7.0 to separate VLDL, LDL, and HDL. TC and TG concentrations in each FPLC fraction were determined using commercial kits, as described above.

The LDLs used for endothelial cell (EC) incubation were from pooled fractions 12–23 separated by FPLC and concentrated using the Ultrafree 15 centrifugal filter device (100K MW; Millipore). The concentrated LDL protein concentration was adjusted to 1 mg/ml.

The VLDL fractions 4–7 and HDL fractions 34–38 (separated by FPLC) were pooled and concentrated using the Ultrafree 15 centrifugal filter device (100K MW; Millipore). We applied 20 μg of total protein of the pooled VLDL, LDL, and HDL fractions to 4–20% SDS-PAGE to detect PCSK9 by Western blot analysis.

### Analysis of active recombinant mouse PCSK9 proteins

Active recombinant mouse PCSK9 (mPCSK9) protein was obtained from Abcam (#Ab167759). This protein is active as measured by its binding ability to biotinylated rhLDLR (Abcam). Our laboratory has tested its biological activity by incubating HepG2 cells with 0, 1, 3, 10, and 20 μg mPCSK9 per well. Western blot analysis of LDLR protein (using #LS-C146979, LSBio; 1:3,000 dilution) showed decreased amounts, indicating that mPCSK9 is biologically active (supplemental Fig. S1)**.** The antibody used to detect mPCSK9 (anti-PCSK9) by Western blot analysis in cell lysates or lipoprotein fractions was from Biolegend (#677502; 1:3,000 dilution).

### Ion mobility measurement of lipoprotein particle concentration

Overnight fasting blood was collected from LDb and LTp mice at 5 months of age using EDTA tubes (n = 3 male mice per genotype). Ion mobility (IM) analysis was employed to directly quantify lipoprotein particles and concentrations over a wide range of sizes from small HDL to large VLDL particles. This IM method measures both the size and concentrations of lipoprotein particle subclasses on the basis of gas-phase differential electric mobility, as previously described (23, 24). Briefly, particle concentrations are measured in 16 size intervals ranging from 7.65 to 54.70 nm. For the analyses reported here, we grouped the following fractions based on size intervals defined for humans: HDL small (HDL 3-2a; 76.5–105 Å), HDL large (HDL 2b; 105.0–145.0 Å), LDL very small (LDL IVc, IVb, IVa, IIIb; 180.0–208 Å), LDL small (LDL IIIa; 208.2–214.1 Å), LDL medium (LDL IIb, IIa; 214.0–224.6 Å), LDL large (LDL I, IDL 2; 224.6–250.0 Å), IDL (IDL 1; 250.0–296.0 Å), and VLDL total (VLDL small, intermediate, large; 296.0–547.0 Å).

### Quantification of atherosclerotic lesions

Atherosclerotic lesions were quantified by two methods: en face and aortic root cross-section. We analyzed 10 male animals per group (LDb and LTp) at 5 months of age for both methods. The en face method measures atherosclerotic lesions throughout the whole aortic tree from the aortic root to the bifurcation of the iliac arteries. The aortic root cross-section quantifies atherosclerotic lesions at the aortic root. Both methods were described in detail by others (25–28) and have been used routinely in our laboratory (16, 17, 19).

#### En face atherosclerotic lesions.

Briefly, mouse aorta was dissected and cleaned to remove adventitial tissues. It was then cut open longitudinally, pinned, and fixed overnight in 10% neutrally buffered formalin, after which it was stained with freshly prepared filtered Oil Red O solution (1.56 mg/ml in methanol). Images of the whole aorta and atherosclerotic lesions were captured and scanned. We used SigmaScan Pro 4.0 imaging software (SPSS Science, Chicago, IL) to quantify the total area of the aorta and the area of atherosclerotic lesions. The results are presented as the ratio of lesions (in square millimeters) divided by the total surface area of the aorta (in square millimeters) in percentage. The images and quantifications of lesions are presented in supplemental Fig. S2A.

#### Aortic root cross-section.

The base of the heart containing the aortic sinus in each mouse was embedded in OCT compound at −80°C. The aortic sinus or aortic root was sequentially sectioned using a cryostat. Once all three aortic valves appeared, serial sections were collected at 5 μm per section. We collected two sections per slide and 9–10 slides per aorta until intact valves were no longer seen. Usually, approximately 18 sections were collected. We fixed and stained every other slide with Oil Red O in a total of three slides of two sections per slide. Six sections per aortic root were used for atherosclerotic lesion measurement. The images were captured by a Zeiss D1M microscope at 25× magnification to cover the whole aortic root section. We carefully drew along the lesion areas that were measured using AxioVision Rel 4.8 software (Zeiss USA, Peabody, MA). The results are presented as area of atherosclerotic lesions (in square microns). The images and quantifications of lesions are presented in supplemental Fig. S2B. Representatives of the continuous sections of LDb and LTp are presented in supplemental Figs. S3A and S3B, respectively.

### Pulse-chase analysis of apoB biosynthesis in mouse primary hepatocytes

Mouse primary hepatocytes (C57BL/6J, LDb, and LTp) were isolated using a procedure modified from that established by David Moore’s laboratory at Baylor College of Medicine, Houston, TX (29). Detailed procedures for extracting primary hepatocytes and performing apoB pulse-chase experiments were previously described (11). The experiments were performed three times with duplicate samples for each time point.

Mouse primary hepatocytes (LDb and LTp) were also incubated with recombinant mPCSK9 (10 μg/ml) for 4 h, followed by apoB pulse-chase experiments, as described. The experiments were performed three times for each time point.

### In vivo macroautophagic flux leupeptin-based assay

We used an in vivo macroautophagic flux assay, described by Haspel et al. (30), to determine autophagy degradation activity in liver. We first intraperitoneally injected either 0.5 ml of PBS or 0.5 ml PBS containing 40 mg/kg leupeptin hemisulfate into 3-month-old male mice: C57BL/6J (PBS, n = 3; leupeptin, n = 3), *Pcsk9*^−/−^ (PBS, n = 3; leupeptin, n = 3), *Ldlr*^−/−^ (PBS, n = 3; leupeptin, n = 3), LDb (PBS, n = 3; leupeptin, n = 3), and LTp (PBS, n = 3; leupeptin, n = 3). After injection, the mice were returned to their cages and had free access to water. At 2 h after injection, the mice were euthanized and the livers were flash-frozen in liquid nitrogen. For liver extracts, we used a 7 ml Dounce homogenizer. We added 3 ml cold homogenization buffer [10 mM Tris (pH 8.0), 5 mM EDTA, 250 mM sucrose, and protease inhibitors] to the liver sample and homogenized with 10 strokes using a loose pestle and 15 strokes with a tight pestle. The crude homogenate was then centrifuged at 700 *g* for 10 min to pellet nuclei and debris. The supernatant was saved as postnuclear S1. To obtain a lysosome-enriched fraction, 2 mg of postnuclear S1 in 1 ml of homogenization buffer was transferred to 1.5 ml microcentrifuge tubes and centrifuged at 20,000 *g* for 30 min at 4°C. The supernatant was labeled as the cytoplasmic fraction. The lysosome-enriched fraction pellet was washed twice with 1 ml of cold homogenization buffer, suspended in 200 μl of 1× sample loading buffer containing 1 mM DTT and directly solubilized in SDS-PAGE buffer to ensure maximum recovery of LC3-II. The experiments were performed at least three times. The results shown are the average of all experiments, expressed as mean ± SEM.

### Western blot analysis of liver homogenates

Liver tissues (50 mg) were homogenized using RIPA buffer (Cell Signaling; #9806) in the presence of protease inhibitors (ThermoFisher; #78441) as previously described (16). Protein concentration was measured by Bradford protein assay (ThermoFisher; #PI23236). Equal amounts of protein (50 μg) were heat-denatured in sample reducing buffer, resolved by SDS-PAGE, and transferred to PVDF membranes (Millipore). The filters were blocked in 5% Blotto for 1 h at room temperature and then incubated overnight at 4°C with primary antibody against Akt-pan-C67E7 and p-ser473-Akt-D9E (Cell Signaling Technology; #4691 and #4060, respectively, at 1:1,000 dilution), AMPKα and p-Thr172-AMPKα (Cell Signaling Technology; #2793 and 2535, respectively, at 1:1000 dilution), ULK1 and p-ser555-ULK1 (Cell Signaling Technology; #8054T and #5869T, respectively, at 1:3,000 dilution), ATG14L (Cell Signaling Technology; #5504S at 1:3,000 dilution), Beclin-1 (BD Bioscience; #612112 at 1:3,000 dilution), LC3 (Novus Biological; #NB100-2220 at 1:3,000 dilution), p62 (Cell Signaling Technology; #5114 at 1:3,000 dilution), and GAPDH-6C5 (Santa Cruz; #SC32233 at 1:5,000 dilution). Immune complexes were detected with IRDye secondary antibodies and the intensity of protein bands was quantified using an Odyssey infrared imaging system (Li-COR).

### Isolation of mouse primary aortic ECs

We isolated mouse primary aortic ECs from C57BL/6J and LDb mice as described by Kobayashi et al. (31). Briefly, the aorta was dissected out from the aortic arch to the abdominal aorta and immersed in 20% FBS-DMEM containing 1,000 U/ml of heparin. We then removed the fat and connective tissues under a stereoscopic microscope and washed the lumen briefly with serum-free DMEM. The lumen was incubated with collagenase type II solution (2 mg/ml in serum-free DMEM) for 30 min at 37°C. ECs were removed from the aorta by flushing with 5 ml of DMEM containing 20% FBS. We collected the ECs by centrifugation at 330 *g* for 5 min. The cells were gently suspended with 2 ml of 20% FBS-DMEM and cultured in a 35 mm collagen type I-coated dish. The cells typically were confluent after 1 week. We stained the cells with anti-CD31 (dilution 1:50) (Neomarker, Fremont, CA) to characterize their morphology.

Primary ECs were plated onto 12-well 0.2% gelatin-coated plates in DMEM endothelial growth medium supplemented with 20% FBS, 2 mM glutamine, 100 U/ml penicillin, 100 μg/ml streptomycin, 1 mM sodium pyruvate, 20 mM HEPES, 1% nonessential amino acids, 50 mM 2-mercaptoethanol, 12 U/ml heparin, and 150 μg/ml EC growth supplement. The next day, the media were switched to serum-free DMEM containing PBS, LDb-LDL (20 μg/ml), or LTp-LDL (20 μg/ml) for 24 h. Cells were collected for RNA and protein extractions.

### Mouse cardiac EC line

The mouse cardiac EC (MCEC) line was purchased from Cedarlane/CELLutions Biosystems (Burlington, NC). This cell line was prepared from microvascular neonatal MCECs that were immortalized with lentiviral vectors carrying the SV40T antigen and human telomerase (32). The cells were characterized as having the phenotypes of ECs, including microtube formation in matrigel, positive staining for CD31, VE-cadherin, and von Willebrand factor-associated antigen, and uptake of DiI-AcLDL. We cultured the cells in DMEM with penicillin/streptomycin, HEPES (10 mmol/l), and 10% FBS in a 0.2% gelatin-coated plate. The cells were seeded onto a 6-well plate at a density of 1 × 10^5^ cells/well. On the next day, the cells were switched to conditions (as described in each experiment) of DMEM-serum free media with control PBS, LDb-LDL (20 μg/ml), LTp-LDL (20 μg/ml), Ldlr^−/−^LDL (20 μg/ml), mPCSK9 (20 μg/ml), or ascending (AC) or descending (DS) LDLs (20 μg/ml) for 24 h. Cells were then collected for RNA and protein extractions, and cell media were collected for analysis of IL-6 and CCL-2 by ELISA, as described below.

### Binding and uptake of DiI-LDL by MCECs

LDLs separated by FPLC from plasma of LDb and LTp mice were concentrated using the Ultrafree 15 centrifugal filter device (100K MW; Millipore) at 500 *g* with a swinging bucket. The final concentration usually reached 1.5 mg/ml. We labeled 0.5 mg of LDL in PBS with 100 μl DiI in DMSO (ThermoFisher, #D282; 3 mg/ml) in a volume of 500 μl at 37°C for 18 h in the dark. The DiI-LDL mixture was then applied to the Ultrafree 15 centrifugal filter device (100K MW) to remove the free DiI by washing the DiI-LDL with 10 ml of PBS twice at 500 *g*. The washed and concentrated DiI-LDLs were used for LDL binding and uptake experiments. The concentration of DiI-LDL was usually 1.0 mg/ml. In parallel with the labeling of LDLs with DiI, a control without LDLs was processed simultaneously using the same procedure. The control was used for EC binding and uptake experiments.

#### LDL binding experiment.

The MCECs were plated onto chamber slides in the DMEM media containing 1% FBS for 24 h prior to the experiment. We then incubated cells with control, DiI-LDb-LDL, or DiI-LTp-LDL (20 μg/ml) in 1% FBS media at 4°C for 30 min to examine LDL binding. The cells were washed with cold PBS three times and fixed in 1% paraformaldehyde in PBS. Cells were examined using the Leica TCS SP5 confocal microscope (Wetzlar, Germany) with a 20× (numerical aperture 0.7) dry objective. We also used a 63× (numerical aperture 1.4) oil objective for higher magnification and resolution.

#### LDL uptake experiment.

The MCECs were plated onto 12-well plates in DMEM media containing 1% FBS for 24 h prior to the experiment. We then incubated cells with control, DiI-LDb-LDL, or DiI-LTp-LDL (20 μg/ml) in 1% FBS media for 4 h at 37°C to measure LDL uptake (internalization). After washing the cells three times with cold PBS, DiI-LDL uptake was examined using a Zeiss Axioskop fluorescence microscope (Zeiss Observer D1m) with a Texas Red filter set.

Flow cytometry was used to sort the cells and to quantify the cells that took up DiI-LDL. We trypsinized the cells that were incubated with DiI-LDL for 4 h at 37°C. The cells were centrifuged followed by washing three times with cold PBS, and resuspended in 1% paraformaldehyde in PBS to produce single cell suspensions. The cells were analyzed by flow cytometry using a Becton-Dickinson FACSCalibur with a Cytek upgrade (BD Biosciences, San Jose, CA). This is a two-laser system; we read the fluorescent signal in the FL2 channel of a 575/26 nm filter. FlowJo CE software was used to quantify the number of cells labeled with DiI. Histograms depicting 10,000 events are representative of three separate experiments.

### Real-time quantitative PCR

Total RNA was extracted from mouse liver, primary ECs, or MCECs using the Qiazol reagent (Qiagen, Valencia, CA), and treated with DNase I (Zymo Research, Inc.) to remove trace amounts of contamination by genomic DNA. The cDNA from each RNA sample was synthesized using a high-capacity cDNA reverse transcription kit supplied by Applied Biosystems (Applied Biosystems, Inc.). The cDNA was subsequently used for real-time quantitative PCR to measure gene expression levels. We used SYBR Green I dye (SYBR Green PCR kit; Bio-Rad) for real-time quantitative PCR with the ABI Prism 7900 sequence detection system (Applied Biosystems) (16, 17). The primers were designed using GenScript Real-time PCR Primer Design software, with primers crossing exon junctions. The nucleotide sequences of each primer were Blast searched against the GenBank database to confirm the uniqueness of each primer. The primers used for this study are shown in supplemental Table S1. The concentration of each primer was optimized to avoid dimer formation. The Ct value of each sample was normalized with the endogenous housekeeping β-actin gene, expressed as relative quantification (RQ). RQ is power of 2^−ΔCt^.

### ELISA quantification of CCL-2, IL-6, and oxidized LDL

We used the mouse IL-6 MAX standard set (Biolegend, San Diego, CA; #431301) and mouse CCL-2 (MCP-1) ELISA Max standard set (Biolegend; #432701) to determine the concentrations of mouse IL-6 and CCL-2, respectively, in cell media. We used a commercial ELISA method to measure mouse plasma oxidized LDL (LSBio; #LS-F13011; supplemental Fig. S4). The detailed procedures are described in the manufacturers’ methods.

### Statistical analysis

We used two-way ANOVA followed by post hoc tests with Bonferroni correction for multiple hypotheses (Fig. 2) to identify changes across plasma lipid components, including TC, CE, FC, PLs, and TG between male LDb and LTp mice at 2, 5, and 8 months of age. The corresponding *P* for each age category and individual lipid are listed on the figure. To determine whether the data was normally distributed, we used a Shapiro-Wilk test with a Bonferroni correction for each set of measurements. This showed no evidence that the values deviated from the normal distribution.

The effects of LDL from three strains (*Ldlr*^−/−^, LDb, and LTp) and other factors, including PBS and mPCSK9 on gene expression levels in MCECs, were first analyzed for all genes studied using two-way ANOVA followed by a post hoc test with Bonferroni correction for multiple hypotheses (Fig. 7B).

Comparison between two groups was performed using two-tailed unpaired *t*-tests with Welch’s correction (GraphPad Prism software, version 5; GraphPad Software, San Diego, CA). A two-tailed *P* < 0.05 was considered to be statistically significant.

## RESULTS

### Deletion of PCSK9 protects LDb mice from developing atherosclerosis

Double KO mice lacking both LDLR and Apobec1 genes (LDb) have elevated plasma levels of apoB100-containing VLDL and LDL and reduced plasma levels of HDL. The dyslipoproteinemia that is produced therefore closely resembles that which occurs in humans. LDb mice develop atherosclerosis spontaneously and lesions appear earlier and are more severe in male than in female mice (11, 16, 17, 19). In the present study, we crossbred Pcsk9^−/−^ mice with LDb mice to generate a triple KO mouse model (LTp).

**Fig. 1.**
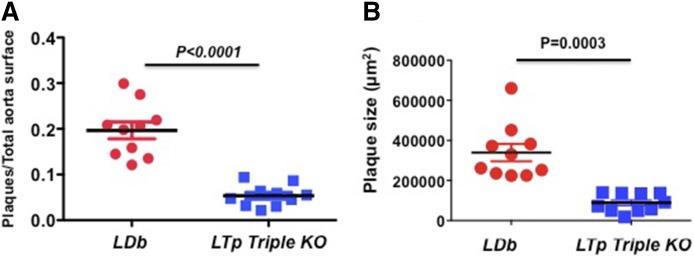
LTp mice develop significantly less atherosclerotic plaque compared with LDb mice. A: En face quantification of aortic atherosclerotic plaque. We quantified the lesions from the aortic arch to the iliac bifurcation of LDb males (n = 10) and LTp males (n = 10) at 5 months of age. The results are calculated as the ratio of aortic surface covered by plaque (in square millimeters) divided by the total surface area of the aorta (in square millimeters) and mean ± SEM is shown. We used two-tailed unpaired *t*-tests with Welch’s correction to analyze the difference between LDb versus LTp (*P* < 0.0001). The image with quantification value of each en face measurement is shown in supplemental Fig. S2A. B: The cross-sections of aortic sinus (n = 6 sections per animal) from each animal (LDb or LTp triple KO) were stained with Oil Red O. The plaque areas (in square microns) of each aortic sinus were quantified using AxioVision release 4.8 software (Zeiss). Each data point represents an average of six sections of each male animal (LDb = 10 and LTp = 10) at 5 months of age and their means ± SEM are shown. We used two-tailed unpaired *t*-tests with Welch’s correction to analyze the difference between LDb versus LTp (*P* = 0.0003). The image with the quantification value of each aortic sinus measurement is shown in supplemental Fig. S2B. The images of continuous image sections are shown in supplemental Figs. S3A and S3B.

LTp triple KO mice were found to have ∼4-fold fewer atherosclerotic lesions compared with *LDb* mice (5.35 ± 0.007% vs. 19.7 ± 0.019%, *P* < 0.0001) at 5 months of age, as determined by en face atherosclerotic measurement (**Fig. 1A**). Additionally, we measured the lesions on the aortic sinus; the total lesions decreased ∼4-fold in LTp triple KO mice, compared with LDb mice (89 ± 14 × 10^3^ μm^2^ vs. 340 ± 43 × 10^3^ μm^2^, *P* = 0.0003) (Fig. 1B). Thus, both measurements demonstrated that deleting the Pcsk9 gene from atherosclerosis-prone LDb mice substantially reduced atherosclerosis, a benefit that was LDLR independent. We next set out to define the mechanism resulting in reduction of atherosclerosis in LTp mice.

### Deletion of PCSK9 results in reductions in plasma lipids and apoB

**Fig. 2.**
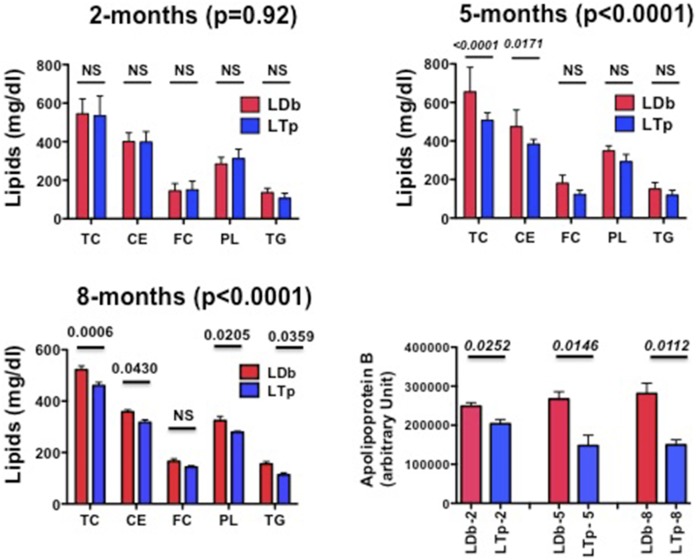
Comparison of plasma lipids and apoB levels in C57BL/6, LDb, and LTp male mice at 2, 5, and 8 months of age. The concentrations of plasma TC, CE, FC, PLs, TG, and apoB in C57BL/6, LDb, and LTp male mice at 2, 5, and 8 months of age (n > 7 animals) are presented as mean ± SD. Statistical analysis on lipids was performed using a two-way ANOVA test, followed by a post hoc test with Bonferroni correction to obtain the *P* for each age category and individual lipids. The *P*s are listed. We used two-tailed unpaired *t*-tests with Welch’s correction to analyze the comparison of apoB between LDb and LTp mice. The *P*s are listed. *P* < 0.05 was considered statistically significant.

We compared plasma lipid and apoB concentrations between LDb and LTp mice at 2, 5, and 8 months of age. By using two-way ANOVA, we could not detect a significant change in the overall lipid levels, including TC, CE, FC, PL, and TG, at 2 months of age (*P* = 0.92, **Fig. 2**). However, by 5 months of age, all lipid levels were decreased significantly in LTp mice compared with LDb mice as determined by two-way ANOVA (TC↓23%, TG↓21%, CE↓19%, FC↓32%, and PL↓16%) and these reductions persisted to 8 months of age. The two-way ANOVA analyses showed highly significant *P* at both 5 and 8 months (*P* < 0.0001, Fig. 2). Some of the individual lipid comparisons also reached statistical significance (Fig. 2). Furthermore, plasma apoB levels in LTp mice decreased at 2 months of age (↓18%) and were reduced further (↓45%) at 5 and 8 months of age in comparison with corresponding LDb mice (*P* = 0.0252, 0.0146, and 0.0112, respectively; Fig. 2). Together, these results show that deletion of PCSK9 decreased plasma lipids and plasma apoB100, irrespective of the presence of LDLR.

### Deletion of PCSK9 decreases apoB biosynthesis rate

**Fig. 3.**
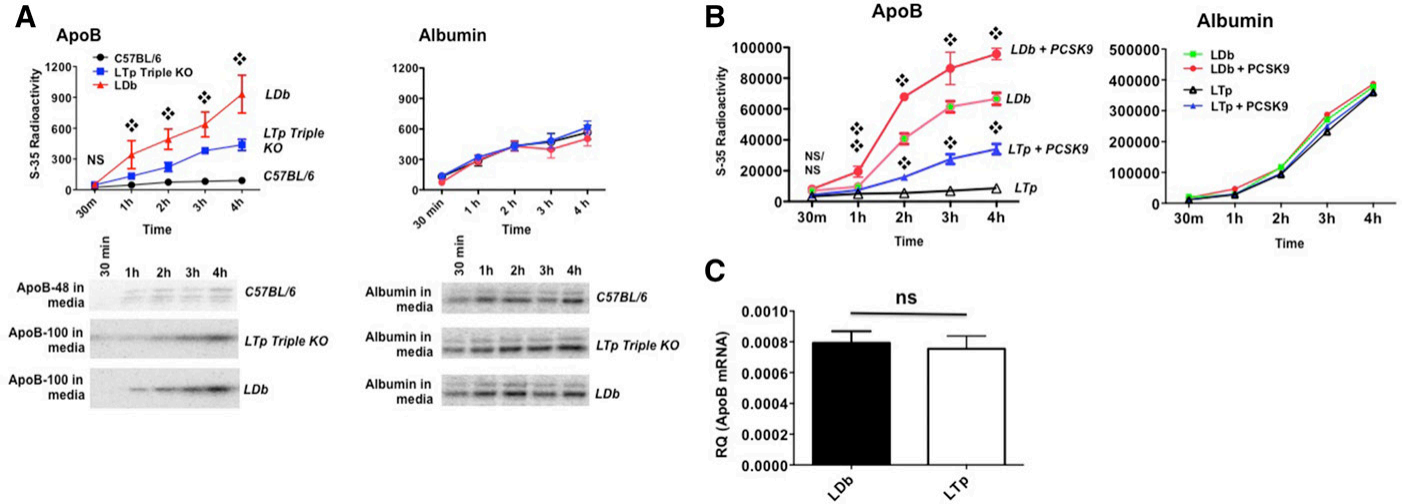
A: The apoB secretion rate is decreased in LTp triple KO mice deficient in PCSK9. Primary hepatocytes were isolated from C57BL/6J, LDb, and LTp mice. Cells were plated onto a 6-well plate coated with mouse type IV collagen. The next day, cells were labeled with ^35^S-methionine/cysteine for 30 min and chased for 30, 60, 120, 180, and 240 min. Cell media were immunoprecipitated with anti-mouse apoB (left) or anti-albumin (right) antibodies and protein A agarose, followed by SDS-PAGE. The bands of apoB100 (LDb and LTp mice) and apoB48 (C57BL/6J mice) were scanned using Typhoon FLA 7000 (GE) and quantified using Quantity One software (Bio-Rad). The results are expressed as total radioactivity in each band (mean ± SD). Statistical analyses were performed using two-tailed unpaired *t*-tests with Welch’s correction. ❖*P* < 0.05, comparing LDb to LTp mice. The experiments were performed three times with duplicate samples for each time point. The radioactivities of apoB were too low to detect in cell lysates. The radioactivities of apoB100 in C57BL/6 were too low to measure in cell media. A representative gel image is shown. B: Recombinant PCSK9 increased the apoB secretion rate in primary hepatocytes from LDb and LTp mice. Primary hepatocytes were isolated from LDb and LTp mice. Cells were plated onto a 6-well plate coated with mouse type IV collagen. The next day, the cells were incubated with 10 μg of recombinant PCSK9 for 4 h and the media were removed and washed. Next, the cells were labeled with ^35^S-methionine/cysteine for 30 min and chased for 30, 60, 120, 180, and 240 min. Cell media were immunoprecipitated with anti-mouse apoB (left) or anti-albumin (right) antibodies and protein A agarose, followed by SDS-PAGE. The bands of apoB100 (LDb and LTp mice) were scanned by Typhoon FLA 7000 (GE) and quantified using ImageQuant TL8.1 software (GE). The experiments were performed three times and the results are expressed as total radioactivity of each band (mean ± SD). Statistical analyses were performed using two-tailed unpaired *t*-tests with Welch’s correction. ❖*P* < 0.05, comparing LDb versus LDb incubated with PCSK9 and LTp versus LTp incubated with PCSK9. C: There is no significant difference in hepatic apoB mRNA levels between LDb and LTp mice. Hepatic apoB mRNA levels in LDb (n = 3) and LTp (n = 3) mice were determined using real-time quantitative RT-PCR. The results are presented as RQ (apoB mRNA normalized with 18S-RNA; mean ± SD). Statistical analyses were performed using two-tailed unpaired *t*-tests with Welch’s correction. The differences are not significant (ns).

Previously, we overexpressed PCSK9 in primary hepatocytes of C57BL/6J, *Ldlr*^−/−^, and LDb mice, resulting in increased apoB synthesis and secretion, as demonstrated by pulse-chase experiments (11). Here, we predicted that the deletion of PCSK9 would have a marked effect on decreasing apoB biosynthesis. We pulsed primary hepatocytes from C57BL/6J, LDb, and LTp mice with ^35^S-methionine/cysteine for 15 min and chased for 30, 60, 120, 180, and 240 min. When compared with LDb mice, the absence of PCSK9 in LTp mice significantly decreased the incorporation of ^35^S-methionine/cysteine into apoB100 secreted into the culture media (**Fig. 3A**). We could only detect apoB48 in control C57BL/6 cells; the radioactivity of the apoB100 band was too low to detect in the culture media. In contrast, there were no differences in the rate of albumin biosynthesis.

We also incubated primary hepatocytes of LDb and LTp mice with recombinant mPCSK9 protein (10 μg/ml) for 4 h, followed by a pulse-chase experiment, as described above. We showed that by incubating mPCSK9 with LTp hepatocytes, there was a significant increase of apoB100 secreted into cell media compared with nontreated LTp hepatocytes (Fig. 3B). Similarly, with incubation of PCSK9 protein with LDb hepatocytes, there was a significant increase of apoB100 in cell media compared with nontreated LDb hepatocytes (Fig. 3B). apoB transcript levels determined by quantitative RT-PCR showed no differences between LDb and LTp mice (Fig. 3C). Together, these results demonstrate that PCSK9 regulates apoB synthesis and secretion in hepatocytes by posttranscriptional mechanisms.

### Deletion of PCSK9 increases autophagy signaling and autophagy flux in mouse liver

**Fig. 4.**
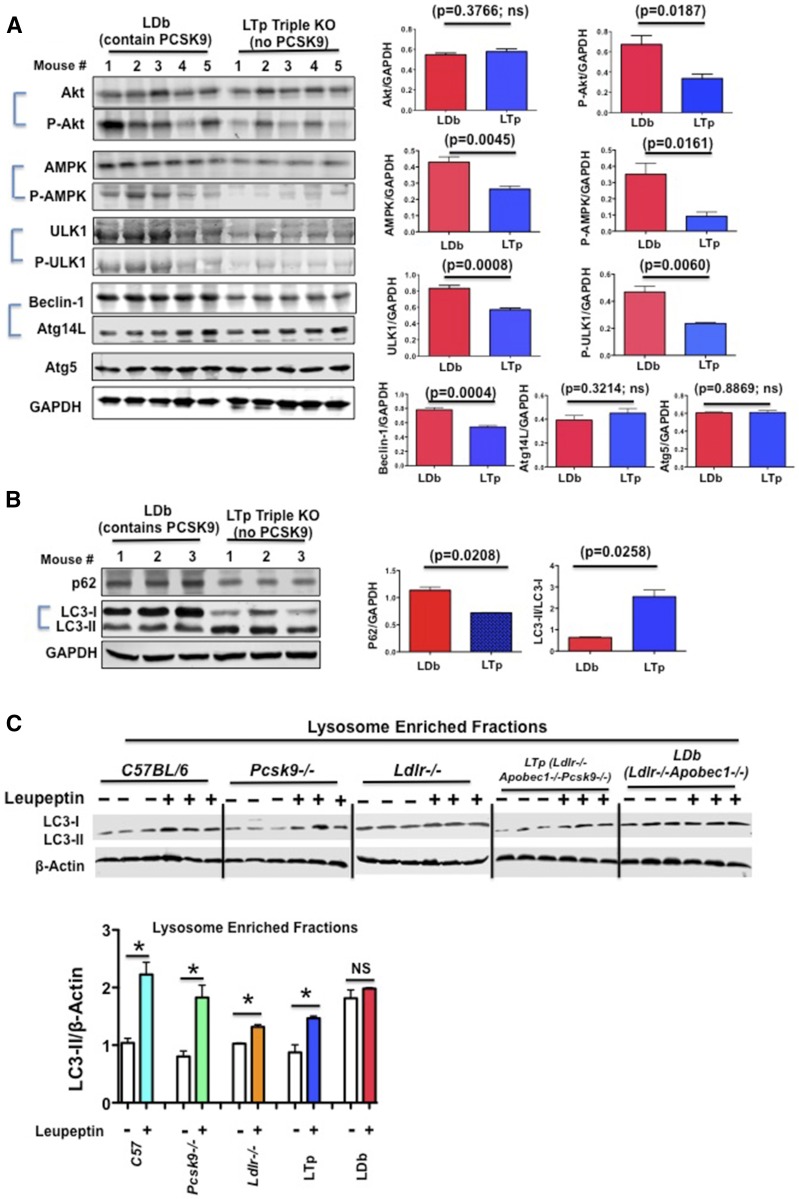
A: Deficiency of PCSK9 in LTp triple KO mice activates the hepatic autophagy signaling pathway. Equal amounts of liver homogenate proteins (50 μg) from overnight-fasted LDb male mice (n = 5) and LTp male mice (n = 5) at 5 months of age were resolved by SDS-PAGE, followed by Western blot to detect Akt, p-Akt, AMPK, p-AMPK, ULK1, p-ULK1, Beclin-1, Atg14L, Atg5, and GAPDH. Each protein was detected and quantified using an Odyssey infrared imaging system (Li-COR). The results are expressed as ratios of intensities of each protein (n = 5)/GAPDH (mean ± SEM). Statistical analyses were performed using two-tailed unpaired *t*-tests with Welch’s correction. ns, not significant. B: Deficiency of PCSK9 in LTp triple KO mice activates hepatic autophagy sequestration function. Equal amounts of liver homogenate proteins (50 μg) from overnight-fasted LDb (n = 3) and LTp (n = 3) male mice at 5 months of age were resolved by SDS-PAGE, followed by Western blot analysis to detect p62 and LC3-I/II. Each protein was detected and quantified using an Odyssey infrared imaging system (Li-COR). The results are expressed as ratios of intensities of each protein (n = 3)/GAPDH and LC3-II/LC3-I (mean ± SEM). Statistical analyses were performed using two-tailed unpaired *t*-tests with Welch’s correction. C: In vivo macroautophagic flux assay. PBS or leupeptin (40 mg/kg body weight) was administered to nonfasted C57BL/6J,* Pcsk9*^−/−^, *Ldlr*^−/−^, LDb, and LTp mice at 3 months of age. At 2 h after injection, livers were collected. LC3-I and LC3-II in liver lysosome-enriched fractions (30 μg/ml) were analyzed by SDS-PAGE, followed by Western blot. LC3-I was mostly not detectable. LC3-II was quantified using the Odyssey infrared imaging system. The results (LC3-II/β-actin) are expressed as mean ± SEM. Statistical analyses were performed using two-tailed unpaired *t*-tests with Welch’s correction. The assays were performed with three mice per genotype. A representative Western blot experimental set of each strain is shown here. *P < 0.05.

We have previously suggested that PCSK9 regulates apoB degradation via modulating autophagy (11). It is known that the autophagy-signaling pathway includes autophagy regulators such as Akt and phosphorylated Akt at Ser 473, AMPK and phosphorylated AMPK at Thr 172, and ULK1 and phosphorylated ULK1 at Ser 555. The Beclin-1 and ATG14L complex initiates the autophagy process. Moreover, p62 shuttles proteins and organelles to autophagosomes for degradation, and the conversion of LC3-I to LC3-II indicates active turnover of the autophagy process. In steady state fasting livers of LDb mice, we found that the levels of phosphorylated Akt were elevated compared with LTp mice (*P* = 0.0187) (**Fig. 4A**). Both AMPK and phosphorylated AMPK (*P* = 0.0045 and 0.0161, respectively), as well as ULK1 and phosphorylated ULK1 (*P* = 0.0008 and 0.0060, respectively) were significantly elevated in LDb mice compared with LTp mice. The levels of Beclin-1 were significantly higher in LDb mice than in LTp mice (*P* = 0.0004), but there were no differences in Atg14L or Atg5. These results demonstrate that PCSK9 might directly or indirectly modulate autophagy signaling. Moreover, the p62 proteins accumulated significantly more in LDb mice than in LTp mice (*P* = 0.0208) (Fig. 4B). In contrast, the conversion of LC3-I to LC3-II was increased more than 4-fold in LTp mice (*P* = 0.0258). Thus, collectively these results point to active induction of the autophagic process in the liver of LTp triple KO mice.

Next, we investigated whether the induction in autophagy in LTp mice was the result of increasing autophagic degradation activity (autophagic flux). We used a method described by Haspel et al. (30) that uses protease inhibition to measure autophagic flux quantitatively in vivo by determining endogenous LC3-II protein turnover. Leupeptin (40 μg/kg body weight), an inhibitor of serine/cysteine proteinase that prevents lysosomal degradation of LC3, was administered to nonfasted C57BL/6J, *Pcsk9*^−/−^, *Ldlr*^−/−^, LDb, and LTp mice. The C57BL/6J, *Pcsk9*^−/−^, and *Ldlr*^−/−^ mice were the controls. When we examined the lysosome-enriched fractions (Fig. 4C), leupeptin increased the accumulation of LC3-II in nonfasting C57BL/6J, *Pcsk9*^−/−^, *Ldlr*^−/−^, and LTp mice (*P* = 0.0019, 0.0048, 0.0011, and 0.0081, respectively) compared with the corresponding PBS-treated mice. In contrast, leupeptin had no significant effect on the accumulation of LC3-II in LDb mice. These results point to dysfunction in the autophagy process in the hyperlipidemic LDb mice, with restoration of hepatic macroautophagy by deletion of the Pcsk9 gene.

### PCSK9 affects the sizes, concentrations, and compositions of plasma lipoprotein particles

**Fig. 5.**
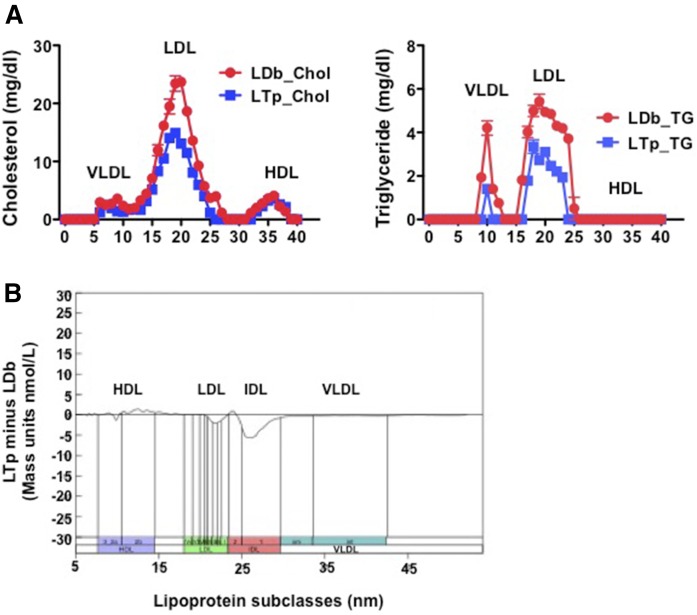
A: Profiles of plasma lipoproteins separated by FPLC. Pooled equal volume (50 μl) plasma samples from LDb (n = 5) and LTp (n = 5) mice at 5 months of age were fractionated by FPLC. The concentration of TC (in milligrams per deciliter) and TG (in milligrams per deciliter) of each fraction was determined. The figure shows the average of three different pooled plasma FPLC runs; each pool contains five animals and the total is 15 animals per group for three separate FPLC runs (LDb, circles; LTp, squares; mean ± SEM). B: Plasma lipoproteins separated using IM analyses. IM analysis was used to separate lipoprotein subclasses from mouse plasma at 5 months of age between LDb (n = 3) and LTp (n = 3) mice. The plot displays the differences in particle concentrations (in nanomoles per liter) across the full lipoprotein size range after conversion of particle to mass concentrations (19); the y axis uses an arbitrary mass scale. The absolute mass concentration of each subclass and statistical analysis are presented in Table 1.

We used FPLC to separate major plasma lipoprotein classes. This revealed markedly decreased concentrations of VLDL and LDL, but not HDL, in LTp mice, compared with LDb mice (**Fig. 5A**). We then used a high-resolution IM method (23, 24, 33) to measure and compare the particle concentrations (in nanomoles per liter) of plasma lipoprotein subfractions of LDb and LTp mice. This revealed that deletion of PCSK9 in LTp mice resulted in significant reductions in distinct species of IDL (250–296 Å, *P* = 0.0220) and medium size LDL (214–220 Å, *P* = 0.0441), but increased large size HDL (105–145 Å, *P* = 0.0295) (Fig. 5B, **Table 1**).

**TABLE 1.** Particle concentrations of lipoprotein subclasses of LTp and LDb mice

Lipoprotein Subclass Concentrations by IM	LTp (nmole/l)	LDb (nmole/l)	LTp Minus LDb (nmole/l)	*P*
HDL small	11,700 ± 634	11,910 ± 541	−202.4 ± 833	ns
HDL large	5,399 ± 284	4,159 ± 243	1240 ± 374	0.0295
LDL very small	69 ± 3.7	70 ± 2.7	−1.0 ± 4.6	ns
LDL small	155 ± 8.8	169 ± 9.0	−13.27 ± 13	ns
LDL medium	192 ± 4.8	228 ± 15	−36.07 ± 16	0.0441
LDL large	515 ± 84	523 ± 77	−7.87 ± 113	ns
IDL	473 ± 35	650 ± 34	−178 ± 49	0.0220
VLDL	39 ± 10	43 ± 11	−3.74 ± 15	ns

Data are expressed as mean ± SEM. All statistical analyses were performed using two-tailed unpaired *t*-tests with Welch’s correction (GraphPad Prism software, version 5). *P* < 0.05 was considered to be statistically significant. ns, not significant.

Next, we measured the compositions of VLDL and LDL that were separated by FPLC from *Ldlr*^−/−^, LTp, and LDb mice (**Table 2**). VLDLs in all strains were enriched with TG (>90%). However, LDb-VLDL contained significantly more protein and PLs than LTp-VLDL. Compared with LDb-LDL, LTp-LDL had significantly less CE (27% vs. 22%, respectively, *P* = 0.0423) and less PL (16% vs. 12%, respectively, *P* = 0.0147), but substantially more FC (7% vs. 21%, respectively, *P* = 0.0033). Similar differences were observed when comparing LDb-LDL to *Ldlr*^−/−^–LDL (Table 2); LDb-LDL contained significantly more CE (*P* = 0.0201) and PLs (*P* = 0.0213), but less FC (*P* = 0.0015). There were no differences in composition between LDL from *Ldlr*^−/−^ and LTp mice. In addition, there was no difference in plasma levels of oxidized LDL between LDb mice and LTp mice (supplemental Fig. S4). Together, these results indicate that inhibition of PCSK9 altered the composition and production of potentially atherogenic lipoprotein species, while increasing levels of potentially protective HDL particles.

**TABLE 2.** VLDL and LDL compositions of mouse plasma lipoproteins separated by FPLC

	TG	CE	FC	PL	Protein
VLDL composition (%)					
*Ldlr*^−/−^	90 ± 0.51	1.91 ± 0.10	0.44 ± 0.19	2.18 ± 0.16	5.68 ± 0.20
LTp	92 ± 0.12	1.81 ± 0.04	0.61 ± 0.12	1.39 ± 0.06	4.24 ± 0.12
LDb	88 ± 1.39	2.84 ± 0.36	1.34 ± 0.29	2.86 ± 0.28	7.86 ± 0.55
LDL composition (%)					
*Ldlr*^−/−^	10 ± 2	23 ± 0.9	17 ± 0.9	13 ± 0.6	37 ± 1.0
LTp	13 ± 1.5	22 ± 1.5	21 ± 2.0	12 ± 0.9	33 ± 1.2
LDb	12 ± 3.8	27 ± 0.9	7 ± 1.0	16 ± 0.6	37 ± 0.7
*P* for LDL					
*Ldlr*^−/−^ versus* LTp*	ns	ns	ns	ns	—
*Ldlr*^−/−^ versus* LDb*	ns	0.0201	0.0045	0.0213	—
*LTp* versus *LDb*	ns	0.0302	0.0246	0.0261	—
*P* for VLDL					
*Ldlr*^−/−^ versus* LTp*	ns	ns	ns	0.0445	0.0087
*Ldlr*^−/−^ versus* LDb*	ns	ns	ns	ns	ns
*LTp* versus *LDb*	ns	ns	ns	0.0358	0.0234

The VLDL and LDL compositions of *Ldlr*^−/−^, LTp, and LDb mice are expressed in percentage of total weight (percent of each lipid ± percent of SEM). *P*s are tabulated. Statistical analyses were performed using two-tailed unpaired *t*-tests with Welch’s correction (GraphPad Prism software, version 5). *P* < 0.05 (two-tailed) was considered to be statistically significant. ns, not significant.

### LDb-LDL is more atherogenic than LTp-LDL

**Fig. 6.**
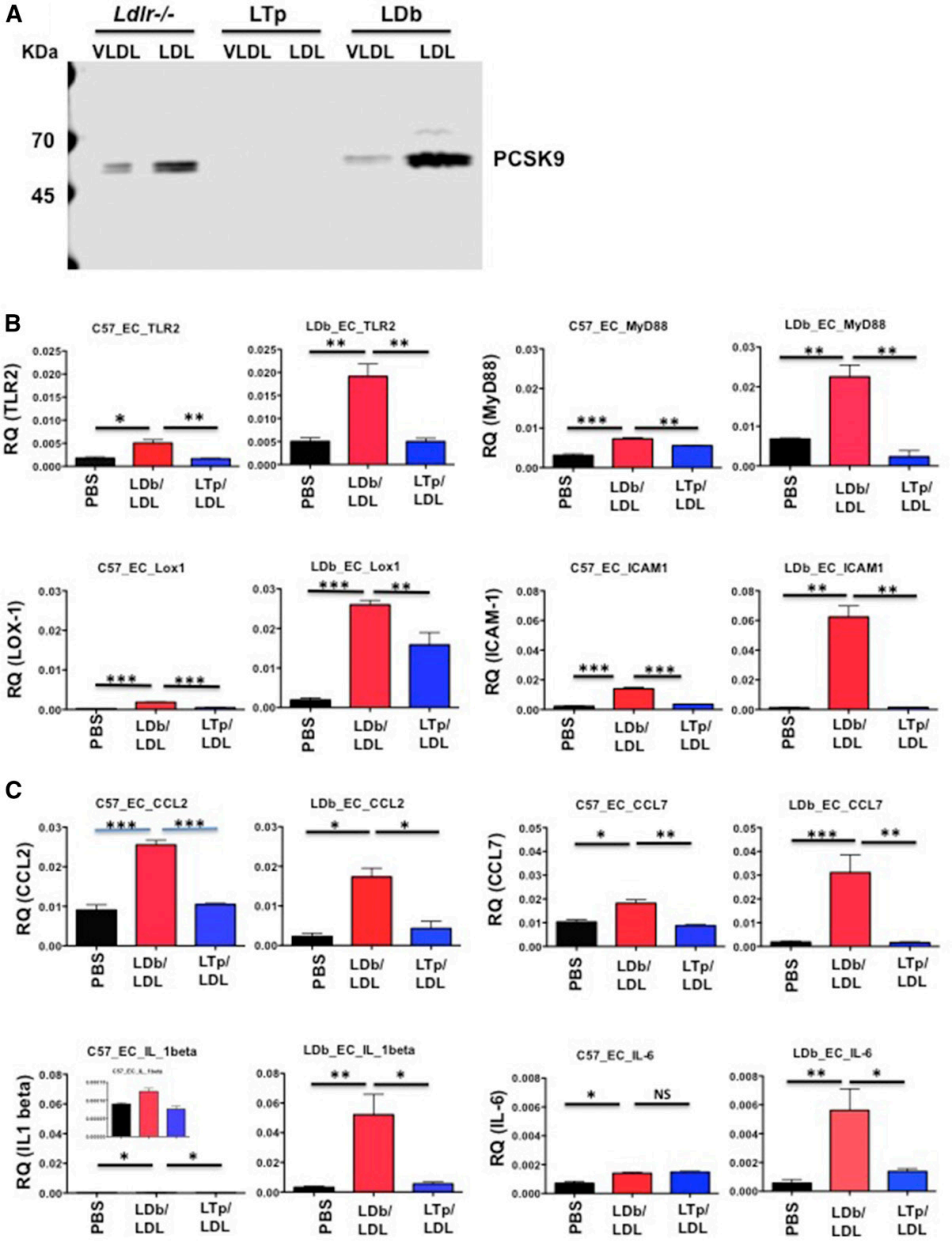

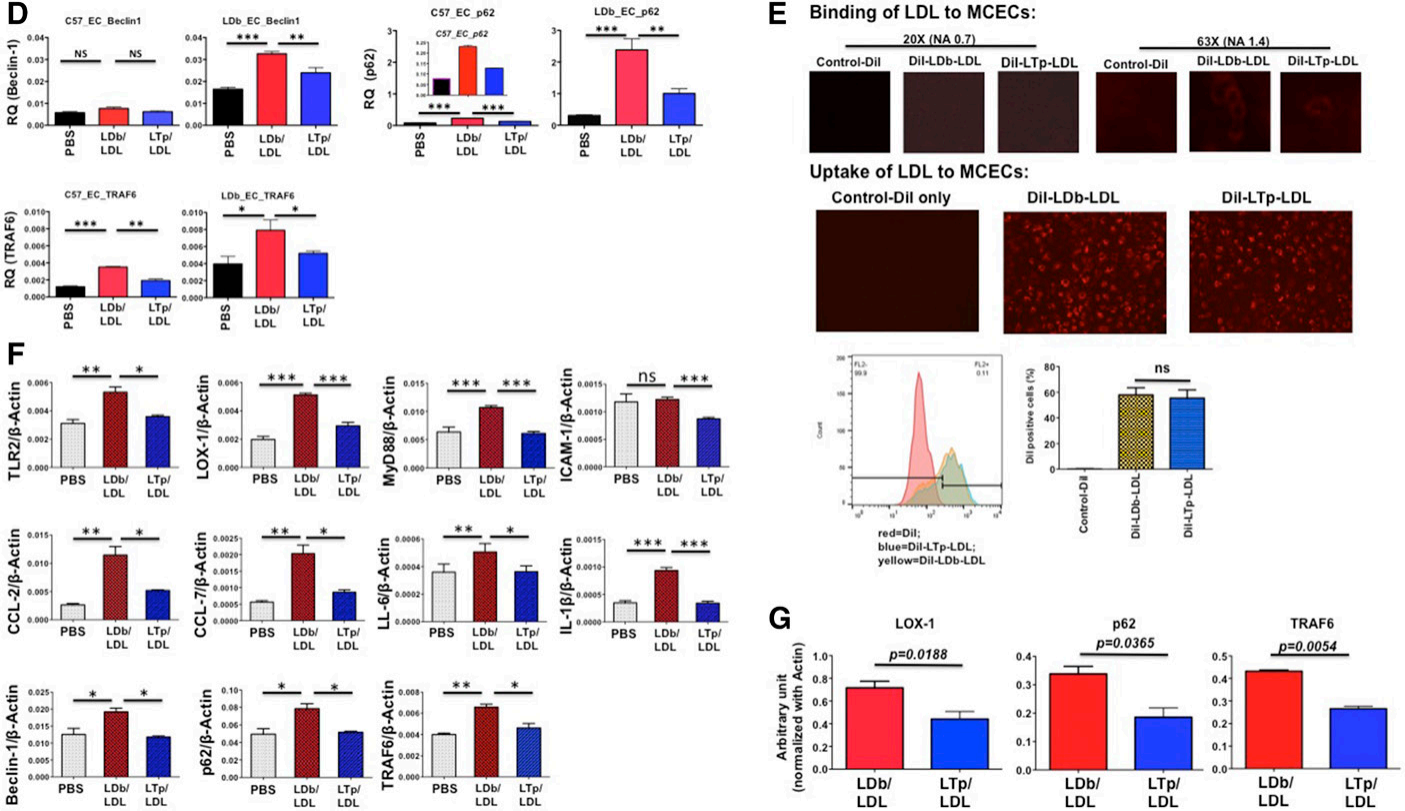

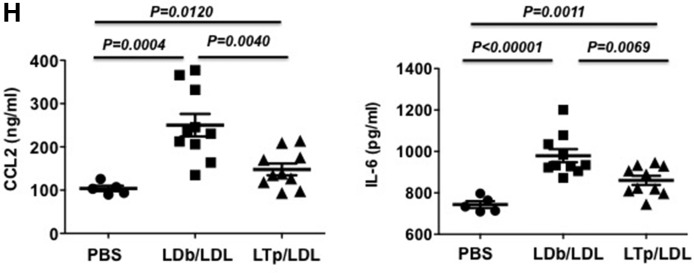
A: PCSK9 is associated with VLDL and LDL from Ldlr^−/−^ and LDb mice. We used FPLC to separate plasma of Ldlr^−/−^, LTp, and LDb mice into lipoproteins. VLDL, LDL, HDL. VLDL and LDL fractions were pooled and concentrated using the Ultrafree 15 centrifugal filter device. We applied 20 μg of proteins to 4–20% SDS-PAGE. PCSK9 was detected by Western blot analysis using anti-PCSK9 (Biolegend; 1:3,000 dilution). The positions of PCSK9 are indicated. There is no detectable PCSK9 in LTp mice. B: LDb-LDL stimulated the gene expression levels of pro-atherosclerosis molecules on aortic primary ECs. Aortic primary ECs were cultured from C57BL/6J (C57_EC) and LDb (LDb_EC) mice and plated onto 6-well plates in duplicate. The next day, each well was incubated with PBS, LDb-LDL (LDL from LDb mice associated with PCSK9, 20 μg/ml), or LTp-LDL (LDL from LTp mice without PCSK9, 20 μg/ml) for 24 h. Cells were collected to extract RNA. The experiment was performed three times. The mRNA levels were measured by real-time quantitative RT-PCR and normalized with β-actin. The results are expressed as RQ, mean ± SEM. Statistical analyses were performed using two-tailed unpaired *t*-tests with Welch’s correction (*P < 0.05, **P < 0.005, ***P < 0.0005). C: LDb-LDL stimulated the gene expression levels of pro-atherosclerosis molecules on aortic primary ECs. Aortic primary ECs were cultured from C57BL/6J (C57_EC) and LDb (LDb_EC) mice and plated onto 6-well plates in duplicate. The next day each well was incubated with PBS, LDb-LDL (LDL from LDb mice associated with PCSK9, 20 μg/ml), or LTp-LDL (LDL from LTp mice without PCSK9, 20 μg/ml) for 24 h. Cells were collected to extract RNA. The experiment was performed three times. The mRNA levels were measured by real-time quantitative RT-PCR and normalized with β-actin. The results are expressed as RQ, mean ± SEM. Statistical analyses were performed using two-tail unpaired *t*-tests with Welch’s correction (*P < 0.05, **P < 0.005, ***P < 0.0005). D: LDb-LDL stimulated the gene expression levels of pro-atherosclerosis molecules on aortic primary ECs. Aortic primary ECs were cultured from C57BL/6J (C57_EC) and LDb (LDb_EC) mice and plated onto 6-well plates in duplicate. The next day, each well was incubated with PBS, LDb-LDL (LDL from LDb mice associated with PCSK9, 20 μg/ml), or LTp-LDL (LDL from LTp mice without PCSK9, 20 μg/ml) for 24 h. Cells were collected to extract RNA. The experiment was performed three times. The mRNA levels were measured by real-time quantitative RT-PCR and normalized with β-actin. The results are expressed as RQ, mean ± SEM. Statistical analyses were performed using two-tailed unpaired *t*-tests with Welch’s correction (*P < 0.05, **P < 0.005, ***P < 0.0005). E: The comparison of binding and uptake of LDb-LDL and LTp-LDL to MCECs. Control DiI, DiI-LDb-LDL, or DiI-LTp-LDL was incubated with MCECs to study the binding and uptake of LDLs to MCECs. The detailed methods are described in the Materials and Methods. Binding of LDLs to MCECs was examined using a confocal microscope. The red dots represent the binding of LDLs to ECs. Both DiI-LDb-LDL and DiI-LTp-LDL bound to ECs after incubation at 4°C for 30 min. The uptake of LDLs to MCECs was carried out at 37°C for 4 h. There were strong uptakes of both LDLs to MCECs. There was no difference in the number of cells taken up by MCECs as determined by flow cytometry (LDb-LDL vs. LTp-LDL; 58 ± 3.2 vs. 56 ± 3.7). ns, not significant. F: LDb-LDL stimulated gene expression levels of pro-atherosclerosis molecules in MCECs. MCECs were plated onto 6-well plates in duplicate. The next day, each well was incubated with PBS, LDb-LDL (LDL from LDb mice associated with PCSK9, 20 μg/ml), or LTp-LDL (LDL from LTp mice without PCSK9, 20 μg/ml) for 24 h. Cells were collected to extract RNA. The experiment was performed three times. The mRNA levels were measured by real-time quantitative RT-PCR and normalized with β-actin. The results are expressed as RQ, mean ± SEM. Statistical analyses were performed using two-tailed unpaired *t*-tests with Welch’s correction (*P < 0.05, **P < 0.005, ***P < 0.0005). G: Protein expression levels of LOX-1, p62, and TRAF6 in MCECs after treatment with either LDb-LDL or LTp-LDL. Protein levels were analyzed by SDS-PAGE followed by Western blot analysis. The results are expressed as mean ± SD. Statistical analyses were performed using two-tailed unpaired *t*-tests with Welch’s correction. H: Protein levels of CCL-2 and IL-6 in the cell media after treatment with PBS (circles), LDb-LDL (squares), or LTp-LDL (triangles). ELISA was used to determine the protein levels of CCL-2 (in nanograms per milliliter) and IL-6 (in picograms per milliliter) in cell media. Individual measurements are shown as well as mean ± SEM. Statistical analyses were performed using two-tailed unpaired *t*-tests with Welch’s correction.

We have shown previously that overexpressed PCSK9 is associated with VLDL and LDL, but not HDL (11). Here, we showed, using FPLC, that endogenous physiological PCSK9 was associated with VLDL and LDL separated from plasma using FPLC in *Ldlr*^−/−^ and LDb mice (**Fig. 6A**).

Because LDLs from LDb and LTp mice differed in composition, we assessed whether LDb-LDL might have differing atherogenic properties than LTp-LDL by comparing the effects of LDb-LDL and LTp-LDL on expression of genes associated with atherosclerosis in primary aortic ECs.

#### Primary ECs with and without LDLR.

We incubated primary ECs obtained from C57BL/6J (C57-ECs, normolipidemia) mice containing LDLR and LDb (LDb-ECs, hyperlipidemia) mice not containing LDLR with LDb-LDL from LDb mice (20 μg/well), LTp-LDL from LTp mice (20 μg/well), or PBS (control untreated) for 24 h. The cells were collected for extraction of RNAs. LDb-LDL, but not LTp-LDL or PBS, markedly induced the expression of *TLR2* and *LOX-1* transcripts in both C57-EC and LDb-EC (Fig. 6B). Similarly, the expression levels of *ICAM-1* and *MyD88* were significantly increased only after incubation with LDb-LDL. With respect to proinflammatory genes (Fig. 6C), only LDb-LDL treatment, but not LTp-LDL or PBS treatment, significantly increased levels of *CCL-2*, *CCL-7*, and *IL-1β* transcripts in both C57-ECs and LDb-ECs. LDb-LDL also significantly increased *IL-6* gene expression in LDb-ECs in comparison with LTp-LDL, but this was not observed for C57-ECs. Finally, in the case of genes that regulate autophagy, LDb-LDL induced expression of *p62* and *TRAF6* in both C57-ECs and LDb-ECs (Fig. 6D), whereas LDb-LDL only induced gene expression levels of *Beclin-1* in LDb-ECs, but not C57-ECs. Taken together, these results demonstrate that LDb-LDL (containing PCSK9) has more atherogenic potential than LTp (not containing PCSK9). The effects of LDb-LDL and LTp-LDL on gene expression in ECs from C57BL/6J and LDb mice were similar; they are not LDLR dependent.

#### MCEC line.

To confirm and extend the findings regarding LDL effects on the expression of atherogenic genes in primary ECs, we performed similar studies using MCECs. MCEC is an immortalized EC line with a stable normal endothelial phenotype that has been used to study the effects of molecules on inflammation (34, 35). We first wanted to establish that this cell line was capable of binding and internalizing LDL particles from our mouse models. We therefore incubated MCECs with DiI-labeled LDb-LDL or LTp-LDL, or DiI-labeled control of DiI. As shown in Fig. 6E, both LDb-LDL and LTp-LDL bound to MCECs and were taken up equally by these cells; the number of cells containing DiI-LDb-LDL and DiI-LTp-LDL were 58 ± 3.2 versus 56 ± 3.7, respectively, as determined by flow cytometry. Thus, both LDb-LDL and LTp-LDL bound to and were internalized by ECs to the same extent.

We then studied the effects of LDb-LDL and LTp-LDL on expression of atherogenic genes in MCECs. MCECs were incubated with LDb-LDL (20 μg/well), LTp-LDL (20 μg/well), or PBS (control). Similar to the results obtained with primary ECs, LDb-LDL significantly stimulated expression of *TLR2* and *LOX-1* genes compared with LTp-LDL (Fig. 6F). LDb-LDL stimulated gene expression of the adaptor protein, *MyD88*, but not *ICAM-1*. LDb-LDL also induced expression of the proinflammatory genes, *CCL-2*, *CCL-7*, *IL-6*, and *IL-1β*. Finally, LDb-LDL activated expression of genes associated with autophagy processes, including *Beclin-1*, *p62*, and *TRAF6*. To confirm the gene expression effects, we performed Western blot analysis for LOX-1, p62, and TRAF6 (Fig. 6G). The protein levels of LOX-1, p62, and TRAF6 were significantly higher in cells incubated with LDb-LDL compared with LTp-LDL, corroborating the gene expression findings. Further, we used ELISA to measure the concentrations of CCL-2 and IL-6 in cell media treated with PBS, LDb-LDL, or LTp-LDL (Fig. 6H). The results showed that both CCL-2 and IL-6 were significantly increased in cells treated with LDb-LDL, compared with those treated with LTp-LDL. Together, the results obtained with MCECs corroborated the findings for primary ECs, suggesting that LDb-LDL has more atherogenic properties than LTp-LDL.

In addition, because our IM analysis (Fig. 5B, **Table 3**) showed that LDb mice had increased amounts of IDL and medium size LDL compared with LTp mice, we thought that larger-sized LDL fractions separated by FPLC might contain some IDL particles. We therefore compared the atherogenic effects of larger (AC) fractions (AC-fractions 12–17) and smaller (DS) fractions (DS-fractions 18–23) of LDLs separated by FPLC between LDb and LTp mice on MCECs. AC-LDLs and DS-LDL had similar amounts of PCSK9, as demonstrated by Western blot analysis (supplemental Fig. S5A). Similar to the results for total LDb-LDL described above, AC-LDb-LDL and DS-LDb-LDL stimulated significantly higher gene expression of *TLR-2*, *LOX-1*, *ICAM-1*, *MyD88*, *CCL-2*, *CCL-7*, *IL-6*, *p62*, and *TRAF6* than that of LTp-LDL. Results for *MyD-88*, *IL-1β*, and *Beclin-1* did not reach statistical significance (supplemental Fig. S5B). Thus, both AC and DS fractions of LDb-LDL had similar effects on induction of proinflammatory and autophagy gene expression.

**TABLE 3.** Statistical analyses of gene expressions in MCECs after treatment with PBS, mPCSK9, LdlrKO-LDL, LTp-LDL, or LDb-LDL

	Genes									
	TLR-2	LOX-1	ICAM-1	MyD88	CCL-2	CCL-7	IL-6	Beclin-1	P62	TRAF6
PBS versus mPCSK9	ns	ns	ns	ns	ns	ns	ns	ns	ns	ns
PBS versus *LdlrKO-LDL*	ns	0.0037	ns	0.0481	ns	0.0033	0.0177	ns	ns	0.0464
PBS versus *LTp-LDL*	ns	ns	ns	ns	0.0061	0.001	0.0414	0.0022	ns	0.0216
PBS versus *LDb-LDL*	0.0141	0.0042	0.0013	0.0123	0.0011	0.0015	0.0102	0.0032	0.0054	0.0027
mPCSK9 versus *LdlrKO-LDL*	ns	0.0014	ns	ns	ns	0.0017	0.0239	0.0213	0.0069	0.0027
mPCSK9 versus *LTp-LDL*	0.0392	ns	ns	ns	0.0027	0.0006	0.0309	0.0059	ns	0.0022
mPCSK9 versus *LDb-LDL*	0.0027	ns	ns	ns	0.0005	0.0012	0.0087	0.0006	0.0001	0.0068
*LdlrKO-LDL* versus *LTp-LDL*	ns	ns	ns	ns	0.0261	ns	ns	ns	ns	ns
*LdlrKO-LDL* versus *LDb-LDL*	ns	ns	ns	ns	0.0007	0.0051	0.0201	0.0022	0.0006	0.0154
*LTp-LDL* versus *LDb-LDL*	0.0233	0.0369	0.0222	0.0255	0.0162	0.0042	0.0201	0.0061	0.0079	0.0188

Comparison of individual gene was performed using two-tailed unpaired *t*-test with Welch’s correction (GraphPad Prism software, version 5). The *P*s are listed and *P* < 0.05 was considered statistically significant. ns, not significant.

### Active recombinant PCSK9 proteins had no effect on proinflammatory gene expression in MCECs

**Fig. 7.**
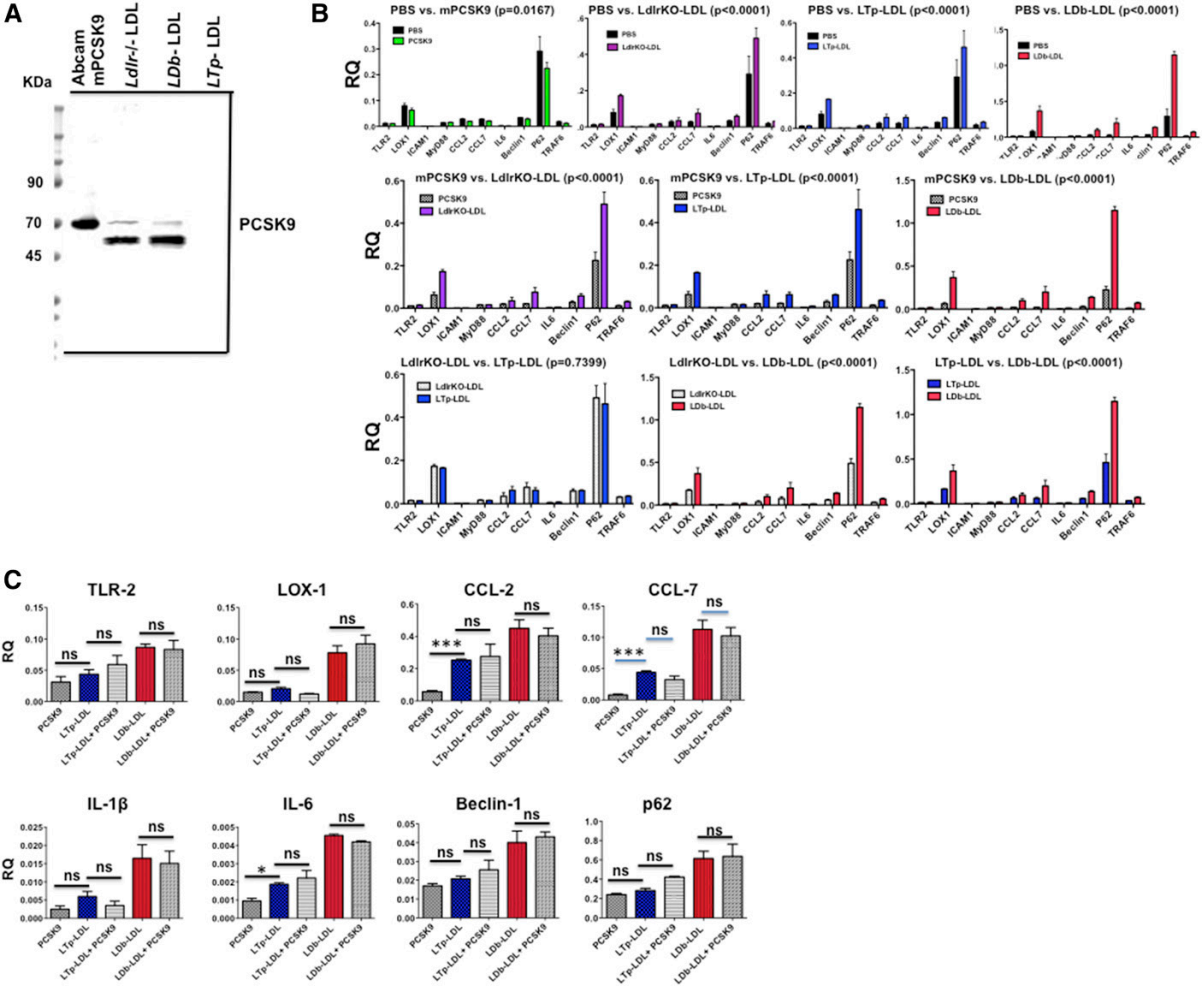
A: The levels of PCSK9 in mPCSK9 and LDLs from *Ldlr*^−/−^, LDb, and LTp mice. The recombinant mPCSK9 (50 ng per lane; Abcam) and LDLs (15 μg per lane) were separated using 4–20% SDS gel electrophoresis, followed by Western blot analysis using anti-PCSK9 (Biolegend; 1:3,000 dilution). The bands of PCSK9 in recombinant mPCSK9 and LDLs from *Ldlr*^−/−^ and LDb mice are shown. LTp-LDL from LTp mice does not have any detectable PCSK9. mPCSK9 (Abcam) has a His-tag in the N terminal and the protein is glycosylated, as indicated by the manufacturer. The expected apparent molecular mass is 66 KDa. B: The effects of mPCSK9 and LDL from *Ldlr*^−/−^, LTp, and LDb mice on proatherogenic and autophagy gene expression in MCECs. MCECs were plated onto 12-well plates in duplicate. The next day each well was incubated with the indicated molecules (20 μg/ml) for 24 h. Cells were collected to extract RNA. mRNA levels were measured by real-time quantitative RT-PCR and normalized with β-actin. The results are expressed as RQ (mean ± SD). We used two-way ANOVA to compare the effect of each factor (PBS, mPCSK9, Ldlr^−/−^LDL, LTp-LDL, and LDb-LDL) on all genes studied as a whole. The *P* of each comparison is listed on the figure. The *P*s of individual gene effects are shown in Table 3. C: The addition of active mPCSK9 to LDb-LDL or LTp-LDL does not yield any synergistic effects on gene expression in MCECs. MCECs were plated onto 12-well plates in duplicate. The next day, each well was incubated with the indicated molecules (20 μg/ml) for 24 h. Cells were collected to extract RNA. The experiment was performed three times. mRNA levels were measured by real-time quantitative RT-PCR and normalized with β-actin. The results are expressed as RQ (mean ± SEM). Statistical analyses were performed using two-tailed unpaired *t*-tests with Welch’s correction (*P < 0.05, **P < 0.005, ***P < 0.0005). ns, not significant.

PCSK9 is secreted into the circulation as a free protein, as well as in association with LDL. Here, we compared the effects on gene expression in MCECs of free PCSK9 (active recombinant mPCSK9; Abcam) with LDLs from *Ldlr*^−/−^, LDb, and LTp mice. Western blot analysis detected PCSK9 protein in recombinant mPCSK9 and in LDLs from *Ldlr*^−/−^ and LDb mice, whereas LDL from LTp mice had no PCSK9 protein (**Fig. 7A**). LDL from *Ldlr*^−/−^ mice was used because both LDLs (*Ldlr*^−/−^ and LTp) had relatively similar composition (Table 2). We incubated MCECs for 24 h with PBS, mPCSK9 (20 μg/ml), or LDLs (20 μg/ml) from *Ldlr*^−/−^, LTp, and LDb mice. Our goal was to define whether free PCSK9 contributed to the induction of gene expression on ECs, whether the effect on gene expression was similar between LTp-LDL and Ldlr^−/−^-LDL, and whether LDb-LDL was more atherogenic than that from LTp or Ldlr^−/−^ mice.

First, we analyzed the effects on overall gene expression changes using a two-way ANOVA test. In comparison to cells treated with PBS control, mPCSK9 significantly decreased expression of all genes studied (*P* = 0.0167). In contrast, cells treated with Ldlr^−/−^-LDL (*P* < 0.0001), LTp-LDL (*P* < 0.0001), and LDb-LDL (*P* < 0.0001) all had significantly increased gene expression (Fig. 7B). There was no significant difference between Ldlr^−/−^-LDL and LTp-LDL treatment (*P* = 0.7399).

Next, when we examined each gene individually, we found that there was no significant difference between the PBS and the mPCSK9 groups (Table 3). Comparison of individual gene expression levels in the other groups showed that most of the significant differences were in genes encoding proinflammatory cytokines (*CCL2*, *CCL7*, and *IL6*), as well as genes encoding proteins associated with autophagy (*Beclin-1*, *p62*, and *TRAF6*). Moreover, individual gene expression analyses showed that LDb-LDL induced significantly higher levels of gene expression than that of LTp-LDL or Ldlr^−/−^-LDL. Together, these analyses suggested that mPCSK9 alone does not contribute to induction of expression of the genes studied.

We then determined whether addition of active recombinant PCSK9 to LDL fractions would have a synergistic effect in modulating gene expression in the ECs. We incubated MCECs with LTp-LDL plus mPCSK9 (20 μg/ml) or LDb-LDL plus mPCSK9 (20 μg/ml). The results (Fig. 7C) showed that addition of free PCSK9 to either LTp-LDL or LDb-LDL yielded no synergistic effects on expression of *TLR-2*, *LOX-1*, *CCL-2*, *CCL-7*, *IL-1β*, *IL-6*, *Beclin-1*, or *p62*. Importantly, this finding suggests that the properties of LDb-LDL, but not PCSK9 per se, induced gene expressions of molecules associated with atherogenesis and autophagy in ECs.

## DISCUSSION

This is the first study to demonstrate that deletion of PCSK9 reduces atherogenesis via mechanisms independent of the LDLR. Deletion of PCSK9 in animals lacking the LDLR reduced the secretion of apoB by increasing the autophagy signaling pathway and autophagy flux in hepatocytes. Not only was production of IDL and medium sized LDL particles reduced, but these particles contained less CE and PLs. When ECs were exposed to these modified lipoprotein particles, they expressed fewer TLR-2 and LOX-1 receptors and less of the adhesion molecule, ICAM-1. Moreover, there was decreased gene expression of the chemotactic factors, CCL-2 (MCP-1) and CCL-7 (MCP-3), that promote monocyte adhesion and infiltration into the vessel wall. LTp-LDL also resulted in decreased levels of IL-6 and IL-1β. Elevated p62 in the vascular wall has been associated with increased atherosclerosis in mice (36). In this study, LTp-LDL reduced the expression of *Beclin-1*, *p62*, and *TRAF6* in ECs, a finding that is consistent with induction of autophagy in these cells. Although these observations do not establish causality, much previous work suggests that some or all of these effects might play a role in the reduction of atherosclerosis that was observed. Recent reports (37, 38) noted that PCSK9 antibodies changed proinflammatory infiltration into arterial walls in FH patients.

**Fig. 8.**
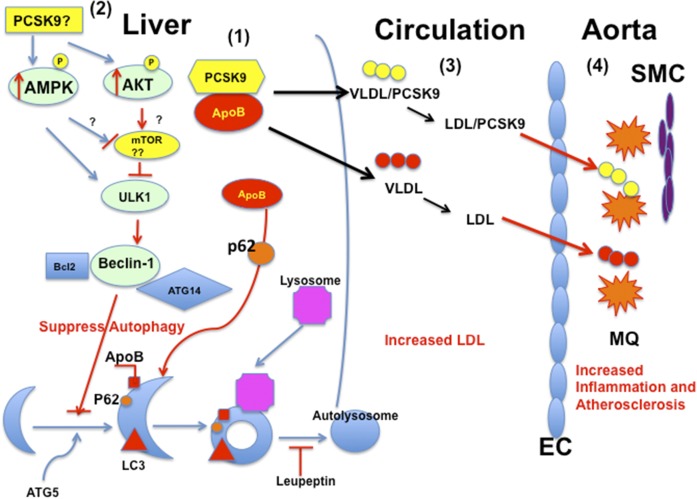
A schematic diagram on regulation of PCSK9 on apoB via modulating the autophagy pathway affecting atherogenesis. **1**: In the liver, PCSK9 interacts with apoB, which inhibits the normal degradation of apoB via the autophagy pathway involving p62. The excess apoB assembles and secretes as VLDL into the circulation. **2**: The regulation of PCSK9 on apoB on assembly and secretion as VLDL intracellularly might activate p-Akt, resulting in suppression of autophagy. On the other hand, this process might induce AMPK and p-AMPK. However, the activation of AMPK and p-AMPK also induces the ULK1 kinase complex and activates Beclin-1 to activate autophagy. In this condition, the regulatory element, Atg14L, in the Beclin-1 complex was not activated. Therefore, the signaling pathway through AMPK did not activate autophagy. Thus, p62 is accumulated in the autophagosome; not degraded, LC3-I is converted to LC3-II inefficiently. Much work is needed to elucidate this complex hypothesis. The role of mTOR in this hypothesis is not confirmed yet. **3**: The VLDLs secreted from liver have different compositions as the result of presence of PCSK9. VLDLs are hydrolyzed to LDLs; the compositions of LDL generated from LDb versus LTp mice are different; LTp-LDL has fewer CEs and PLs. **4**: LDb-LDL and LTp-LDL induce different activations on ECs and immune cells, leading to inflammatory reaction of macrophages (MQs) and migration/proliferation of smooth muscle cells (SMCs). This is the initiation of atherogenesis.

apoB has been shown to accumulate within cytoplasmic lipid droplets where proteosomal and autophagic degradation converge (39, 40). Autophagy has been recognized as a dynamic regulatory process that degrades apoB (41–43). By overexpressing PCSK9, we have shown that PCSK9 interacts with apoB, resulting in inhibition of the degradation pathway of apoB via an autophagic mechanism (11). The results of the present study suggest that PCSK9 may play a role in increasing Akt, AMPK, and ULK1 protein expression, which could influence the autophagy-signaling pathway. Akt has many downstream targets, including mTOR. Akt can activate mTOR by inhibiting the ULK1 kinase complex, resulting in suppression of autophagy (44). In the presence of PCSK9, AMPK and p-AMPK proteins are activated. AMPK can activate the ULK1 complex, resulting in activation of Beclin-1. However, our results suggested that this activation did not lead to activation of autophagy because the regulatory molecule, Atg14L, in the Beclin-1 complex was not activated (45) (Fig. 8). We found that PCSK9 led to increased levels of p62 with low levels of conversion of LC3-I to LC3-II, indicating a dysfunction in the autophagy process possibly involving sequestration (Fig. 8). In vivo leupeptin measurements confirmed that the absence of PCSK9 activates autophagic flux in these animals. Taken together, our results suggested that PCSK9 regulates apoB secretion via affecting autophagy. Questions remain as to whether PCSK9 regulates this fundamental cellular function in hepatocytes directly, and/or whether it interacts with apoB to shuttle apoB for secretion rather than degradation via the autophagy pathway. The potential role of PCSK9-mediated autophagy or PCSK9-apoB-autophagy is novel and a potentially important fundamental insight because this process could have an important influence on lipoprotein metabolism and atherogenesis.

PCSK9 was initially reported by Fan et al. (46) to be associated with LDL and HDL, but not with VLDL. By overexpressing human PCSK9 in C57BL/6J, *Ldlr*^−/−^, and LDb mice, we found that PCSK9 was associated with VLDL and LDL, but not with HDL (11). The current study shows endogenous physiological PCSK9 is associated with VLDL and LDL. Our findings are consistent with a mechanism whereby this association results from direct interaction of PCSK9 with apoB intracellularly (11), thus reducing degradation of apoB via autophagy and increasing apoB secretion and plasma levels of both VLDL and LDL.

Using plasma from human PCSK9 transgenic mice, Tavori et al. (12) showed that PCSK9 is co-immunoprecipitated with apoB100 and apoB48. Using both iodixanol density gradient ultracentrifugation and FPLC methods to separate lipoproteins, they detected PCSK9 in the LDL fraction, but not in the VLDL fraction (12). Similarly, Kosenko et al. (47), using iodixanol density gradient ultracentrifugation and FPLC, observed that PCSK9 was associated with the LDL fraction from human plasma. While previous studies (12, 47) using FPLC failed to detect the association of PCSK9 with VLDL that we observed, our use of two Superose 6 columns in series achieved better separation of VLDL and LDL than was reported in the earlier studies (12, 47); although due to the size heterogeneity of VLDL and LDL particles, there is some overlap in their size distributions.

We showed here that LDLs from *Ldlr*^−/−^ mice and LTp mice had LDLs with similar composition, and both LDLs induced similar levels of responses from ECs. In contrast, LDL from LDb mice had elevated amounts of CE and PLs, and produced higher levels of proatherogenic and proinflammatory responses. These LDLs also provoked malfunction of autophagic processes in ECs. Thus, evidence from this study suggests that lipid-enriched LDL resulting from the presence of PCSK9, but not PCSK9 alone, may have an atherogenic effect on ECs. Interestingly, the LDL compositions from *Ldlr*^−/−^ and LTp mice were similar and the effects of these two LDLs on responses to ECs were also similar, which may explain the observation by Denis et al. (9) that PCSK9 does not play a role in atherogenesis in *Ldlr*^−/−^ mice.

Autophagy in vascular ECs protects against many pathophysiological stimuli such as modified LDL and many other factors (48). Thus, dysfunction of autophagy is implicated in atherosclerosis development. Razani et al. (36) shows that autophagy becomes dysfunctional with elevated p62 levels as the atherosclerotic plaque develops. In our study, we showed that endothelial gene expression of molecules associated with autophagy, including Beclin-1, p62, and TRAF6, were increased after treatment with hyperlipidemic LDb-LDL, but not LTp-LDL. It has been shown that different lipid species play various roles in the regulation of autophagosomal biogenesis, both as membrane constituents and as signaling platforms (49). CE accumulation in macrophages has been shown to inhibit macroautophagy in ECs (50–52). PLs, such as phosphatidylethanolamine or phosphatidic acid, are important factors at several stages of autophagy (49). Thus, it is possible that increased levels of CE and PLs in LDb-LDL, but not LTp-LDL, may contribute to impaired autophagy in ECs.

We found that LTp mice lacking PCSK9 developed significantly fewer atherosclerotic lesions than LDb mice. We only backcrossed these mice to C57BL/6J for six generations and, thus, there was residual heterozygosity that would have increased the variance of the results. Because residual variants from each original genotype are expected to either have no impact on the phenotype of interest or impact the phenotype randomly in both directions. Thus, our results are not biased, but instead are strengthened, by the presence of residual heterozygosity.

Although our study was focused on the effects of PCSK9 on hepatic apoB secretion and its potential relation to atherogenesis, PCSK9 is also substantially expressed in the small intestine in mice and in the human LoVo-C5 and CaCo-2 cell lines (53, 54), and administration of recombinant PCSK9 has been found to decrease LDLR content in the small intestine in Pcsk9^−/−^ mice (55) and in CaCo-2 cells (13). Moreover, Le May et al. (53) have shown that Pcsk9^−/−^ mice have decreased apoB100 and apoB48 synthesis in mesenteric lymph compared with wild-type mice. These Pcsk9^−/−^ mice also secrete larger chylomicrons with higher hepatic clearance rate. In vitro studies in CaCo-2 cells have shown a positive correlation between intestinal PCSK9 expression levels and apoB secretion (14, 53, 55). Levy et al. (13) found that regulation of apoB synthesis by PCSK9 in CaCo-2 cells is independent of the LDLR. In this regard, Rashid et al. (14) demonstrated that PCSK9 increases intestinal microsomal TG transfer protein mRNA levels independently of the LDLR. Together, these findings indicate that intestinal PCSK9 may be linked with the production of apoB in the small intestine, as well as intestinal chylomicron assembly. Moreover, deficiency of intestinal PCSK9 has been found to reduce postprandial hypertriglyceridemia by increasing the clearance of TG-rich lipoproteins (53, 56). *Apobec1*^−/−^ mice, producing only apoB100, secrete larger chylomicron particles than those of wild-type mice (57, 58). In the absence of PCSK9, these larger chylomicrons might be subject to faster hepatic clearance (53). These observations raise the possibility that changes in intestinal as well as hepatic lipoprotein metabolism may contribute to our finding of decreased atherogenesis in LTp mice.

In summary, our findings indicate that hepatic reduction of PCSK9 expression could affect atherogenesis by modulating the levels and properties of apoB-containing lipoproteins that are independent of PCSK9’s effects on cell surface LDLRs. This suggests that interventions such as siRNA (59), anti-sense RNA, ribozymes, or gene editing, that are targeted at PCSK9 gene expression in the liver might benefit cardiovascular disease even more than can be achieved by administration of PCSK9 monoclonal antibodies.

## Supplementary Material

Supplemental Data
