# Supplementary material for: PCSK9 deficiency reduces atherosclerosis, apolipoprotein B secretion, and endothelial dysfunction
Source: J Lipid Res. 2017 Nov 27;59(2):207–23. doi: 10.1194/jlr.M078360 (PMC5794417; doi:10.1194/jlr.M078360)
Supplement: Supplemental Data [file supp_59_2_207__index.html]

PCSK9 deficiency reduces atherosclerosis, apolipoprotein B secretion and endothelial dysfunction — PCSK9 deficiency reduces atherosclerosis, apolipoprotein B secretion, and endothelial dysfunction — Supplemental Data 

# PCSK9 deficiency reduces atherosclerosis, apolipoprotein B secretion, and endothelial dysfunction

## Supplemental Data

- Supplemental Table STable1 and Supplemental S figures (.pdf, 14.4 MB) - Supplemental Table S1; Supplemental Figure S1; Supplemental Figure S2A; Supplemental Figure S2B; Supplemental Figure S3A; Supplemental Figure S3B; Supplemental Figure S4; Supplemental Figure S5A; Supplemental Figure S5B
