# Supplementary material for: PCSK9 deficiency reduces atherosclerosis, apolipoprotein B secretion, and endothelial dysfunction
Source: J Lipid Res. 2017 Nov 27;59(2):207–23. doi: 10.1194/jlr.M078360 (PMC5794417; doi:10.1194/jlr.M078360)
Supplement: Supplemental Data [file 10.1194_M078360_jlr.M078360-1.pdf]

Supplementary Material

PCSK9 deficiency reduces atherosclerosis, apolipoprotein B secretion and endothelial dysfunction

Hua Sun[1], PhD; Ronald M. Krauss[4], MD; Jeffrey T. Chang[2, 3], PhD; Ba-Bie Teng[1, 3], PhD

[1] Research Center for Human Genetics, The Brown Foundation Institute of Molecular Medicine, and

[2]Department of Integrative Biology and Pharmacology, The University of Texas Health Science Center at Houston (UTHealth), Houston, TX

[3] The University of Texas MD Anderson Cancer Center UTHealth Graduate School of Biomedical Sciences (GSBS).

[4] Children's Hospital Oakland Research Institute, 5700 Martin Luther King Jr. Way, Oakland, CA 94609

Correspondence:

Ba-Bie Teng, PhD, Research Center for Human Genetics, Suite SRB 530D, The Brown Foundation Institute of Molecular Medicine, The University of Texas Health Science Center at Houston, 1825 Pressler St., Houston, TX

Telephone number: 713-500-2443

Fax number: 713-500-2447

Email: babie.teng@uth.tmc.edu

Running title: PCSK9 mediates atherogenesis

Supplemental Table S1: Sequences and concentrations of primers and probes

Gene Symbol	Forward primers (5-3), optimized concentration	Reverse primers (5-3), optimized concentration
**Srebp-1a** NM_011480	5′ CGGCTCTGGAACAGACACTG (2 **μM)**	5′ GAAGTCACTGTCTTGGTTGTTG (2 **μM)**
**Srebp1-c** NM_011480	5' ATCGGCGCGGAAGCTGTCGGGGTAGCG (2 **μM)**	5' GAAGTCACTGTCTTGGTTGTTG (2 **μM)**
**Srebp2** NM_033218	5' GTTCTGGAGACCATGGAG (5 **μM)**	5' AAACAAATCAGGGAACTCTC (5 **μM)**
**HMGCoA reductase** NM_008255	5' CTTTCAGAAACGAACTGTAGCTCAC (2 **μM)**	5' CTAGTGGAAGATGAATGGACATGAT (2 **μM)**
**HMGCoA synthase** NM_145942	5' CAGCCATTTGTTACAGCTTATTCTC (2 **μM)**	5' TCTTTTTAATTGCCACATATTATTTTAGAA (2 **μM)**
**Acc** NM_133904	5' TTATCTCTGGAGAACCTCTCTAATGG (2 **μM)**	5' AGACACTTAGCAAGAGCAAAAATGA (2 **μM)**
**Fas** NM_007988	5' AGCACTGCCTTCGGTTCAGTC (2 **μM)**	5' AAGAGCTGTGGAGGCCACTTG (2 **μM)**
**Scd1** NM_009127	5' CTGCAGGTTGTGCTAGATGGGATGG (2 **μM)**	5' GCCTGGGGTCTTTGGTAAGTAGGC (2 **μM)**
**Gpat** NM_008149	5' CCTCTCAGTGGTAGTGGATACTCTGT (1 **μM)**	5' GTGACCTTCGATTATGCGATCAT (1 **μM)**
Ppap D84376	5' CCTCTCAGTGGTAGTGGATACTCTGT (2 **μM)**	5′ GAATATTCCTCGCTGGAAGG (2 **μM)**
Cds1 NM_173370	5′ TGCAGTTCCTCATTCGCTAC (2 **μM)**	5′ GCGTCCATGCGAACATATAG (2 **μM)**
**Dgat1** NM_012079	5' GGCATTCACAGCAATGATGGC (2 **μM)**	5' CCACACAGCTGCATTGCCATA (2 **μM)**
**Dgat2**	5′ TGTCACCTGGCTCAACAGATC (2 **μM)**	5′ TATCAGCCAGCAGTCAGTGCA (2 **μM)**
**TLR2** NM_011905	5′ TTGTTCCCTGTGTTGCTGGT (1 **μM)**	5′ ACAAAGTGGTTGTCGCCTGCT (1 **μM)**
**Lox1** NM_138648	5′ CTGGATTGGATTGCATCGGAA (2 **μM)**	5′ CAGCTCCGTCTTGAAGGTATG (2 **μM)**
**MyD88** NM_010851	5′ AGAGCTGCTGGCCTTGTTAG (2 **μM)**	5′ TTCTCGGACTCCTGGTTCTG (2 **μM)**
**Icam1** NM_010493	5′ GCCTCCGGACTTTCGATCTT (2 **μM)**	5′ GTAGACTGTTAAGGTCCTCTGCGT (2 **μM)**
**Ccl-2** NM_011333	5′ CTCAGCCAGATGCAGTTAACGCCC (1 **μM)**	5′ GGTGCTGAAGACCTTAGGGCAGAT (1 **μM)**
**Ccl-7** NM_013654	5′ CTCATAGCCGCTGCTTTCAGCATC (1 **μM)**	5′ GTCTAAGTATGCTATAGCCTCCTC (1 **μM)**
**IL-6** NM_031168	5′ CTCTGGGAAATCGTGGAAATG (1 **μM)**	5′ AAGTGCATCATCGTTGTTCATACA (1 **μM)**
**IL-1beta** NM_008361	5′ AAGGAGAACCAAGCAACGACAAAA (1 **μM)**	5′ TGGGGAACTCTGCAGACTCAAACT (1 **μM)**
**Beclin-1** NM_019584	5′ ACCAGCTGGACACTCAGCTCAA (2 **μM)**	5′ GCAGCTGCTCACTGTCATCCTC (2 **μM)**
**P62** NM_011018	5′ TGAAACATGGACACTTTGGCT (2 **μM)**	5′ ACATTGGGATCTTCTGGTGGA (2 **μM)**
**Traf6** NM_009424	5′ GCAGTGAAAGATGACAGCGTGA (2 **μM)**	5′ TCCCGTAAAGCCATCAAGCA (2 **μM)**
**Beta-Actin** NM_007393	5′ TGGAATCCTGTGGCATCCATGAAAC (1 **μM)**	5′ TAAAACGCAGCTCAGTAACAGTCCG (1 **μM)**
**mApoB** NM_009693	5' ATGTACTAATTGCCATAGATAGTGCCA (0.25 **μM)**	5' TCGCGTATGTCTCAAGTTGAGAG (0.25 **μM)**
	Probe: 6FAM-ATCAACTTCAATGAAAAA-MGBNFQ (0.12 **μM)**	
**18S (X00686)**	5' TAACGAACGAGACTCTGGCAT (0.17 **μM)**	5' CGGACATCTAAGGGCATCACAG (0.17 **μM)**
	Probe: 6FAM-TGGCTGAACGCCACTTGTCCCTCTAA-TAMRA (0.11 **μM)**	

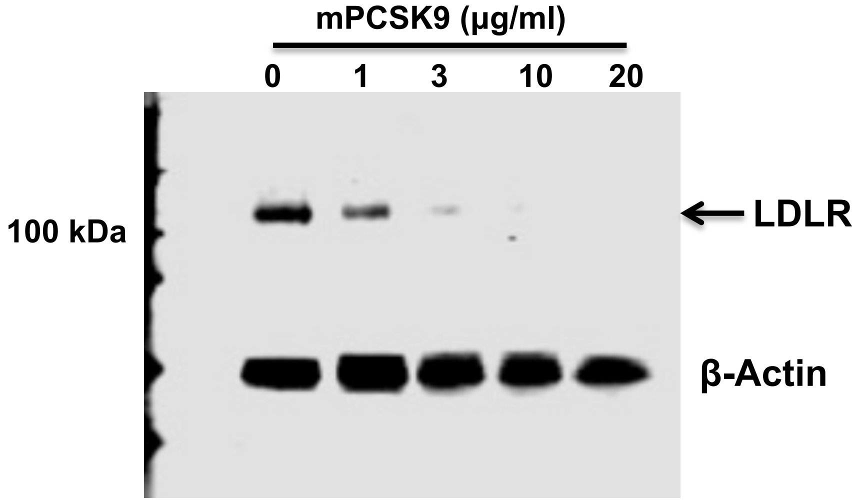

Supplemental Figure S1: The recombinant mPCSK9 is active. HepG2 cells were plated onto 6-well plate. The cells were incubated with indicated concentrations of recombinant mPCSK9 (Abcam) for 24 h. The next day, cell lysates were collected. We applied equal amount of cellular protein (100 µg) to 4-20% SDS-PAGE, followed by Western blot analysis. Primary antibody is rabbit anti-LDLR (Bethyl,1:3000 dilution). The secondary antibody is anti-rabbit Alexa 680 (In Vitrogen, 1:10,000 dilution). The position of LDLR is indicated.

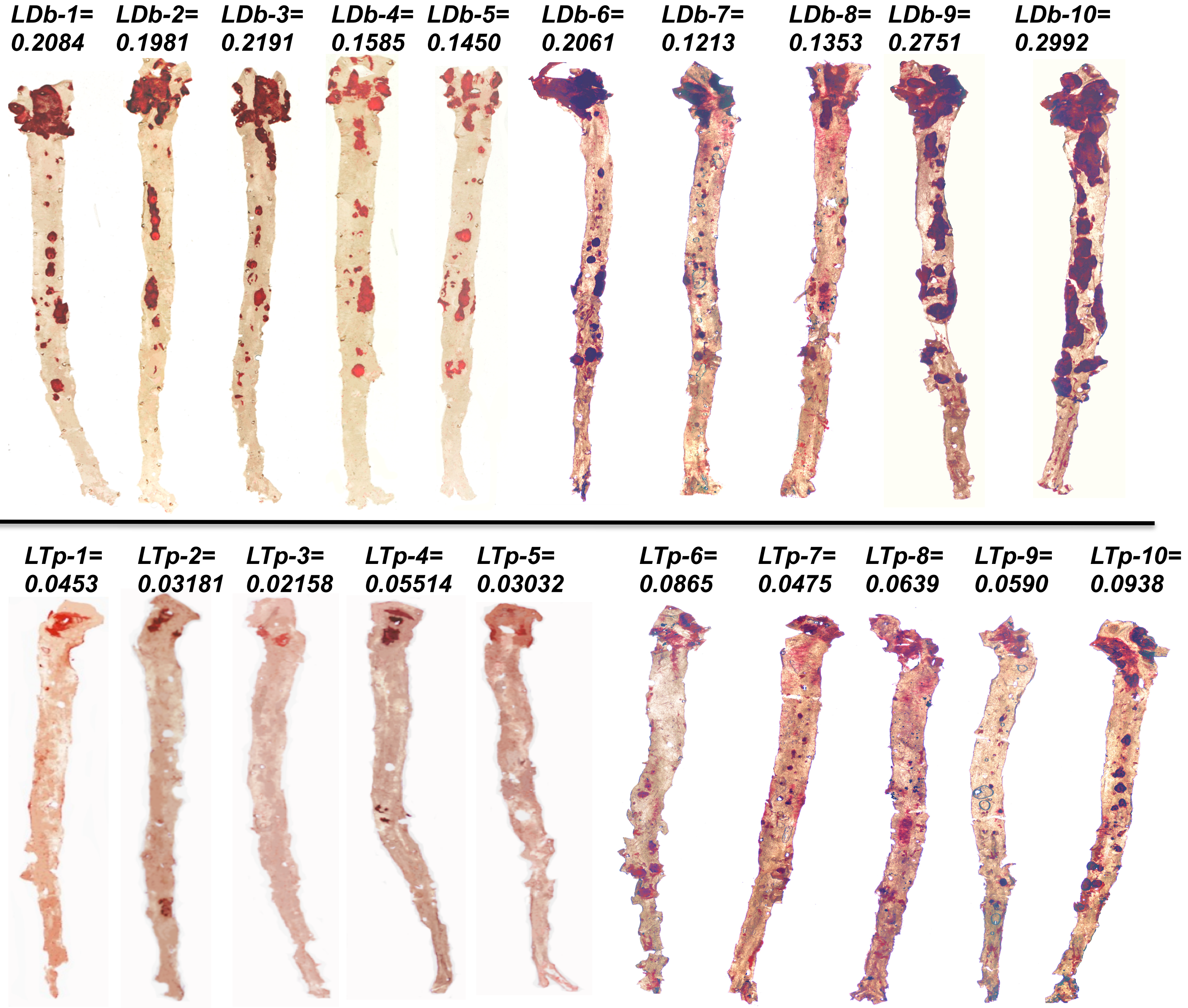
LDb-1= 0.2084
LDb-2= 0.1981
LDb-3= 0.2191
LDb-4= 0.1585
LDb-5= 0.1450
LDb-6= 0.2061
LDb-7= 0.1213
LDb-8= 0.1353
LDb-9= 0.2751
LDb-10= 0.2992
LTp-1= 0.0453
LTp-2= 0.03181
LTp-3= 0.02158
LTp-4= 0.05514
LTp-5= 0.03032
LTp-6= 0.0865
LTp-7= 0.0475
LTp-8= 0.0639
LTp-9= 0.0590
LTp-10= 0.0938

Supplemental Figure S2A. ***En face* quantification of aorta of LDb and LTp mice**

The result of each aorta is listed, which is calculated as the ratio of aortic surface covered by plaques (mm^2) divided by the total surface area of the aorta (mm^2).

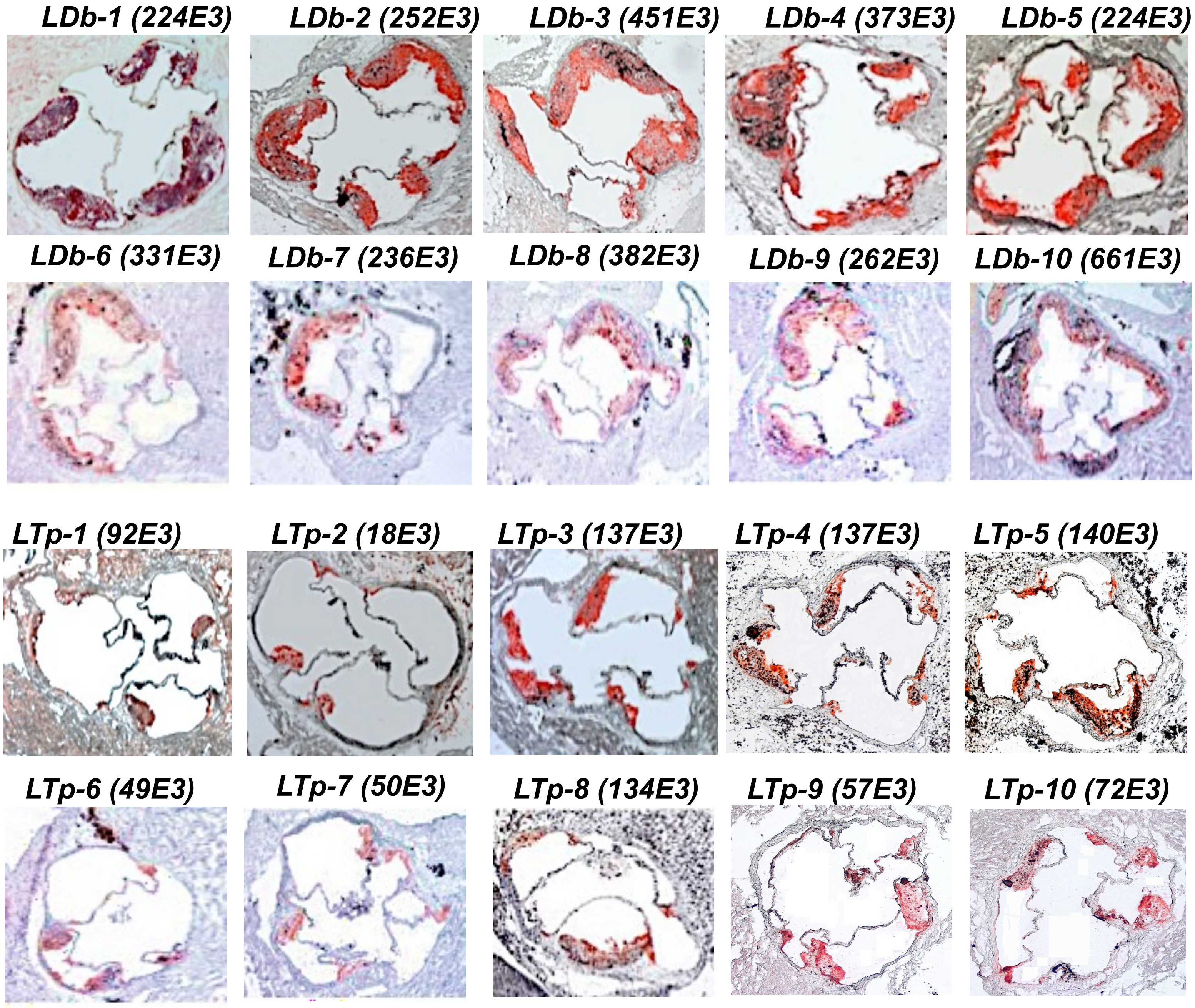
LDb-1 (224E3)
LDb-2 (252E3)
LDb-3 (451E3)
LDb-4 (373E3)
LDb-5 (224E3)
LDb-6 (331E3)
LDb-7 (236E3)
LDb-8 (382E3)
LDb-9 (262E3)
LDb-10 (661E3)
LTp-1 (92E3)
LTp-2 (18E3)
LTp-3 (137E3)
LTp-4 (137E3)
LTp-5 (140E3)
LTp-6 (49E3)
LTp-7 (50E3)
LTp-8 (134E3)
LTp-9 (57E3)
LTp-10 (72E3)

Supplemental Figure S2B. Measurement of atherosclerotic lesions on Aortic Sinus from LDb and LTp mice

The measurements of the aortic sinus plaque area are shown as μm^2 X 10^3 (μm^2 E3).

Supplemental Figure S3A. Continuous Aortic Sinus Sections at 5μm/section.
LDb mouse, male, 5-months of age.

1st slide➔

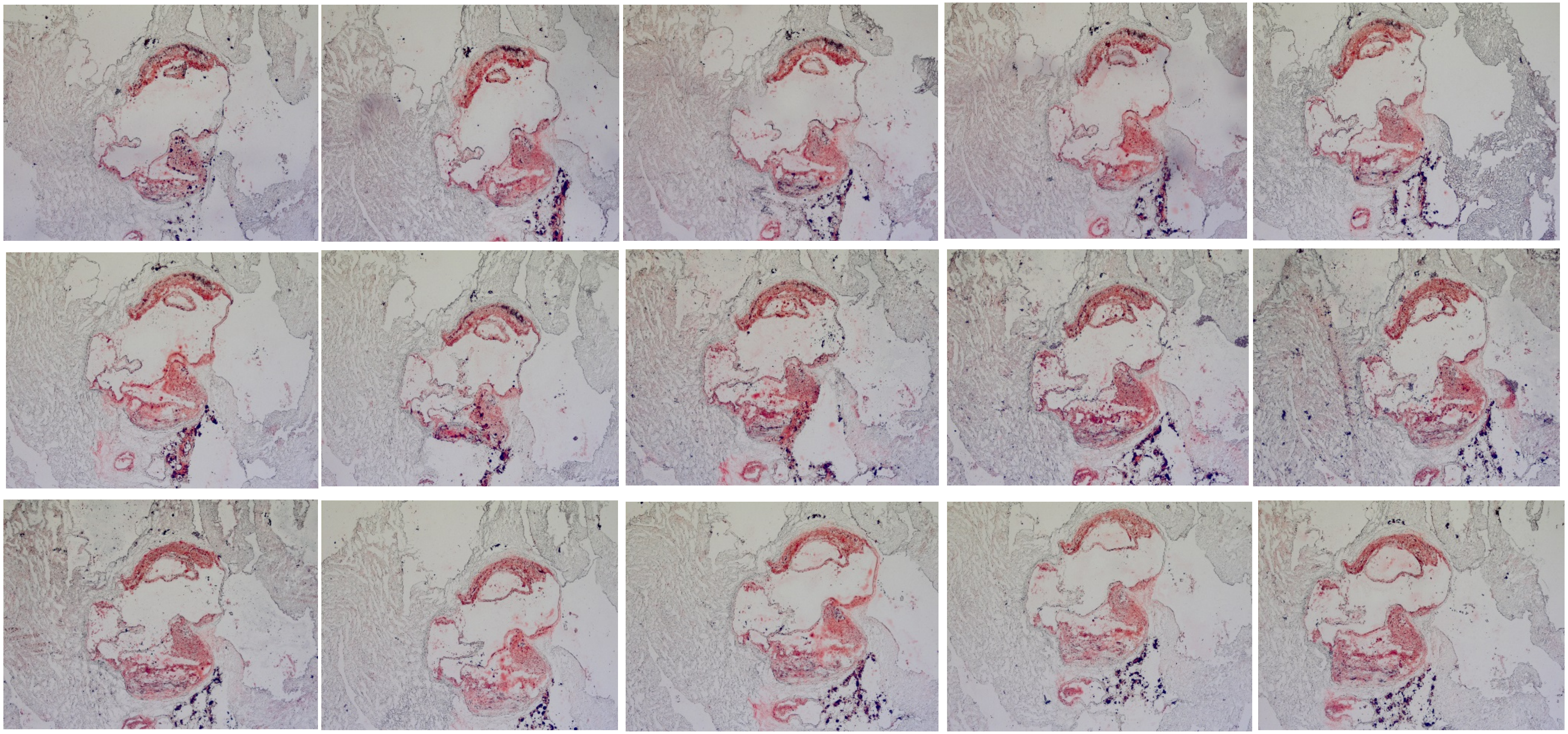

Continue to #16 ➔

Supplementary Figure S3A. Continuous Aortic Sinus Sections at 5μm/section.
LDb mouse, male, 5-months of age.

#16

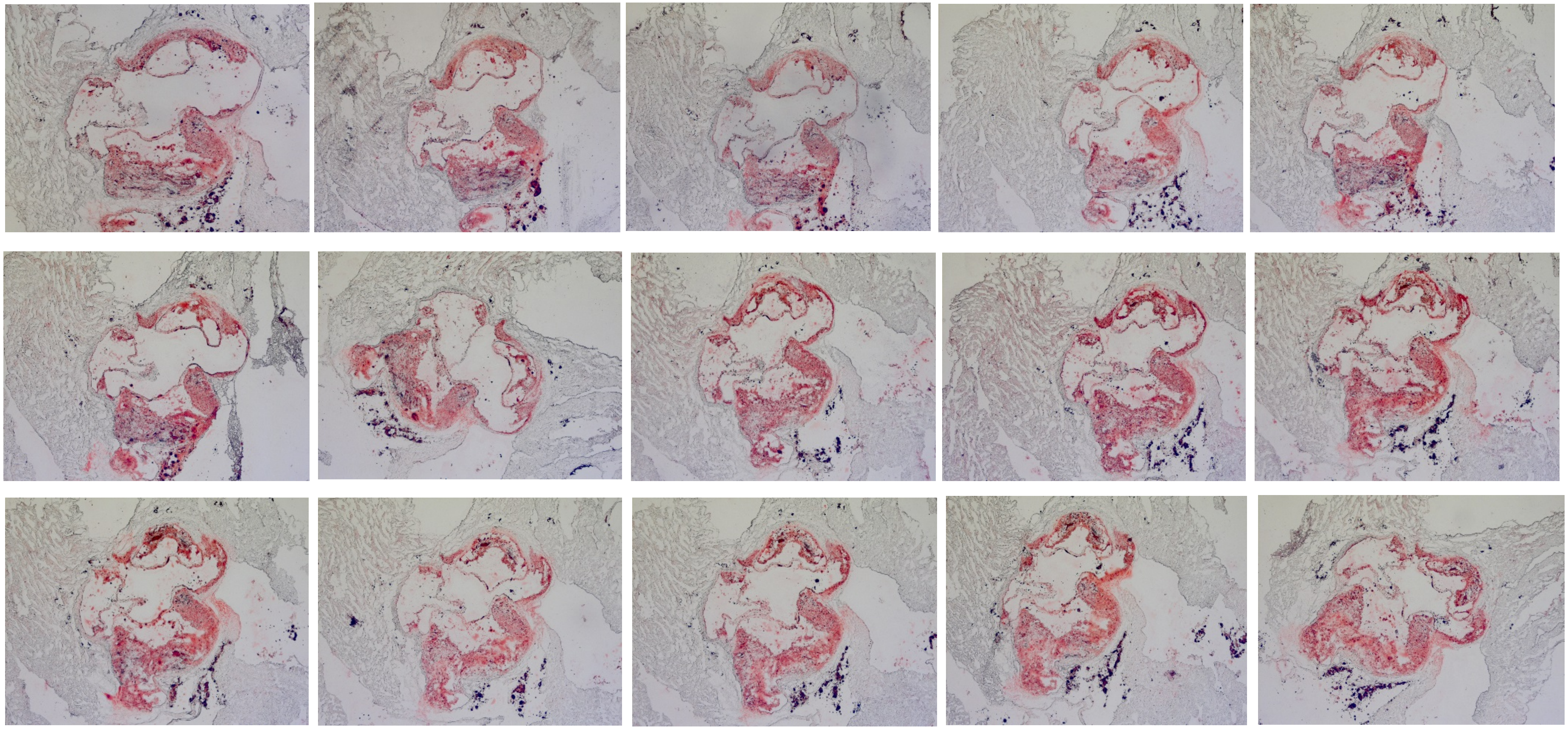

Continue to #31 ➔

Supplemental Figure S3A. Continuous Aortic Sinus Sections at 5µm/section.
LDb mouse, male, 5-months of age.

#31

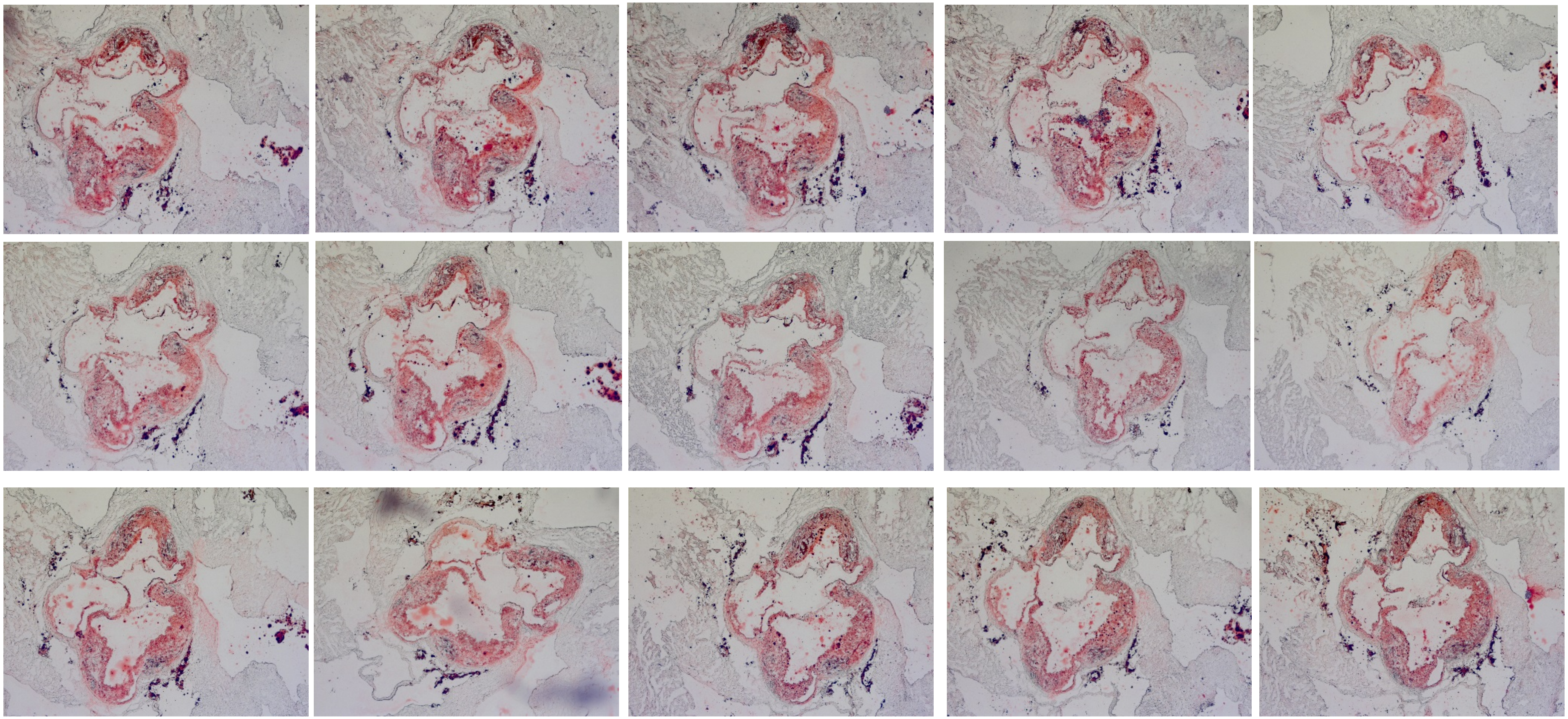

Continue to #46 →

Supplemental Figure S3A. Continuous Aortic Sinus Sections at 5μm/section.
LDb mouse, male, 5-months of age.

#46

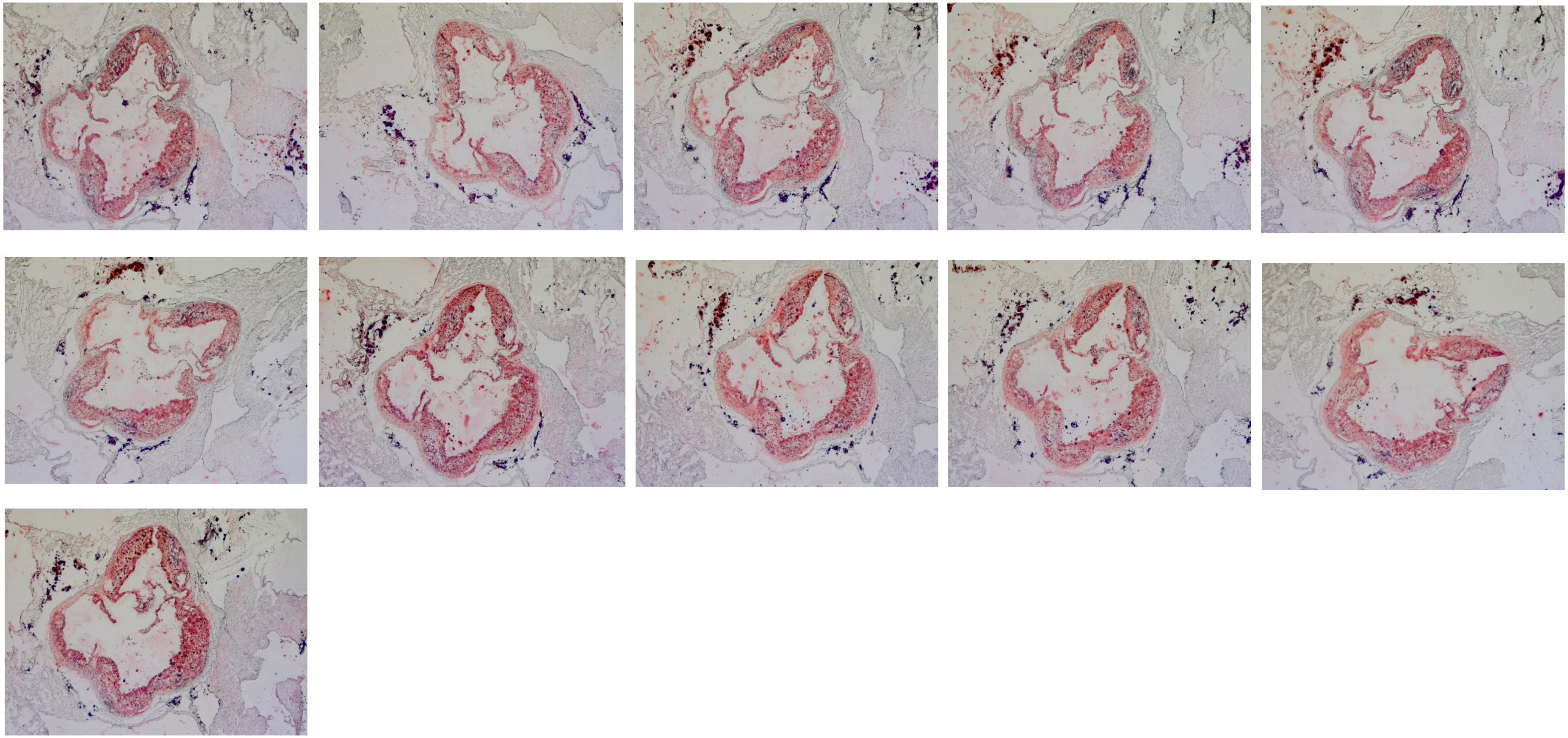

End, last section #56

Supplemental Figure S3B. Continuous Aortic Sinus Sections at 5µm/section.
LTp (Ldlr-/-Apobec1-/-Pcsk9-/-) mouse, male, 5-months of age.

1st slide➔

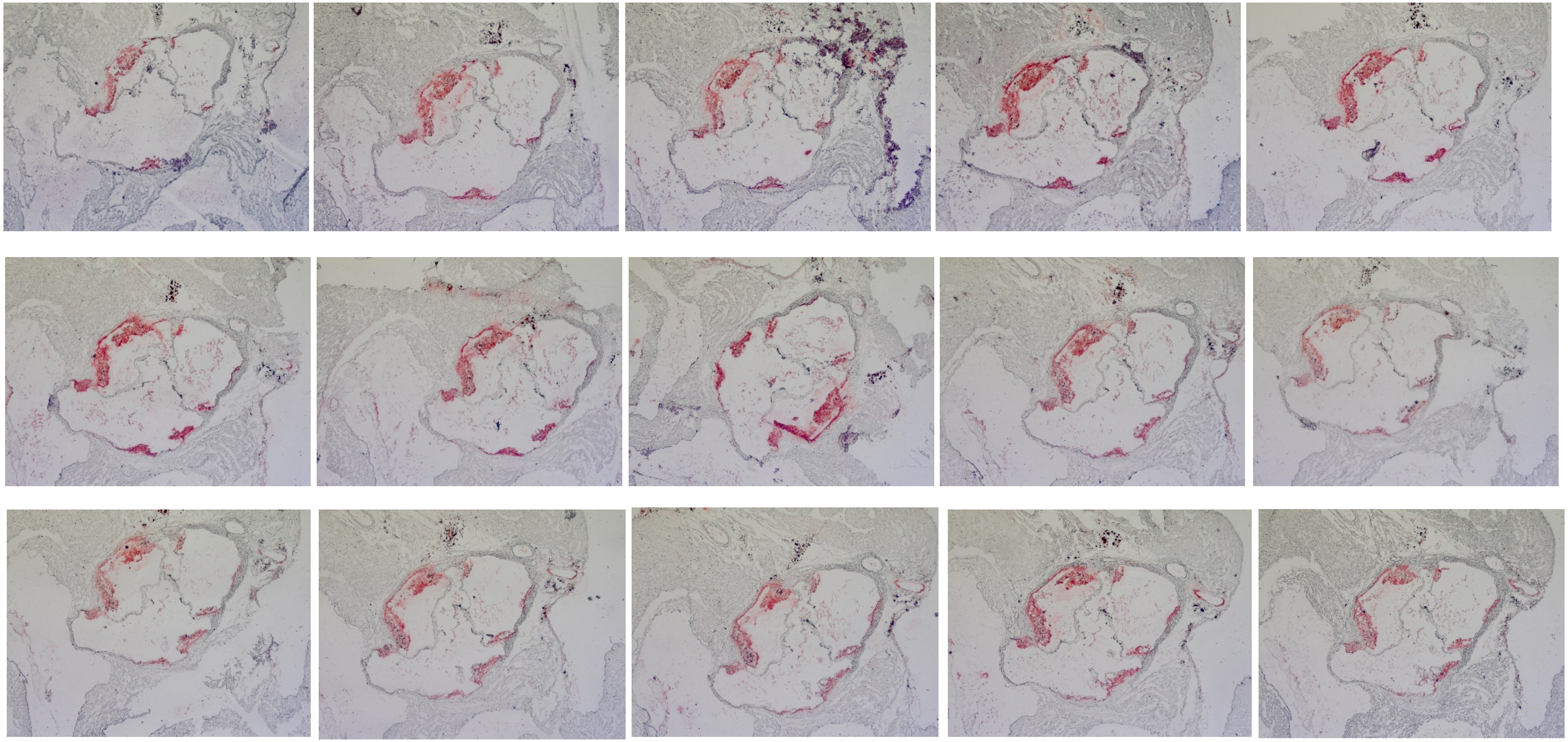

Continue to #16 ➔

Supplemental Figure S3B. Continuous Aortic Sinus Sections at 5μm/section.
LTp (Ldlr-/-Apobec1-/-Pcsk9-/-) mouse, male, 5-months of age.

LTp, #16

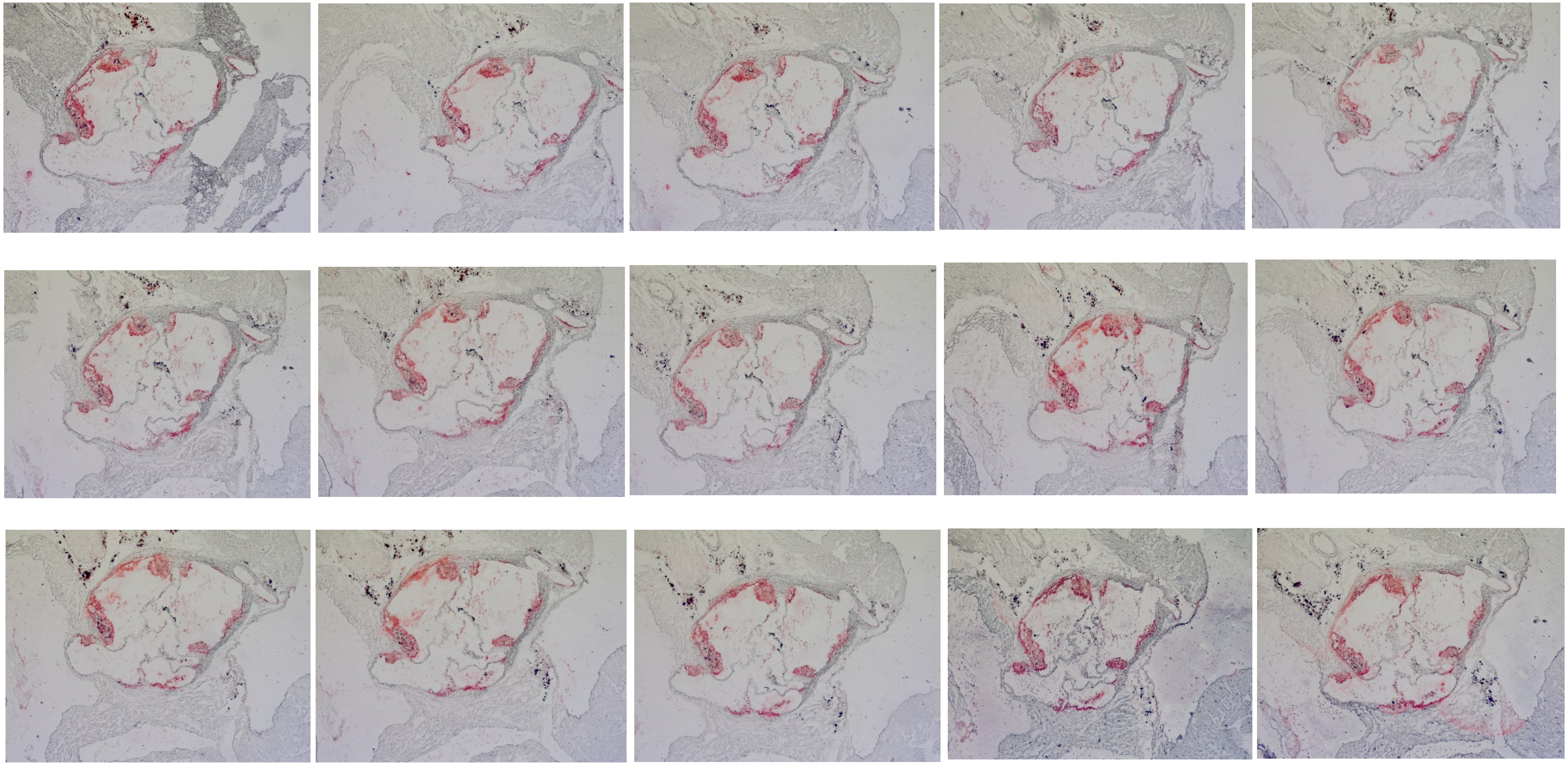

Continue to #31 ➔

Supplemental Figure S3B. Continuous Aortic Sinus Sections at 5µm/section.
LTp (Ldlr-/-Apobec1-/-Pcsk9-/-) mouse, male, 5-months of age.

LTp, #31

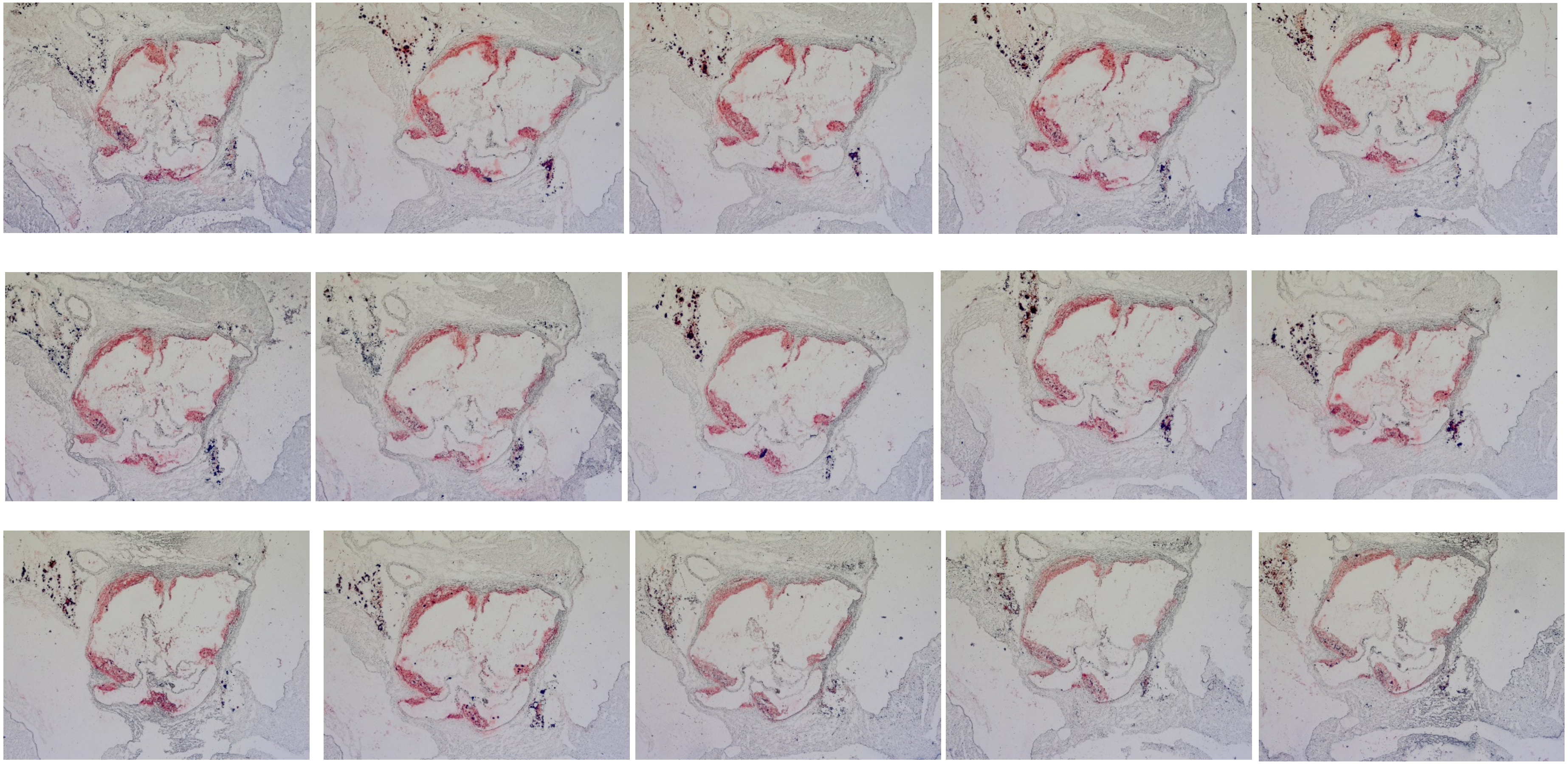

End, Last section, LTp

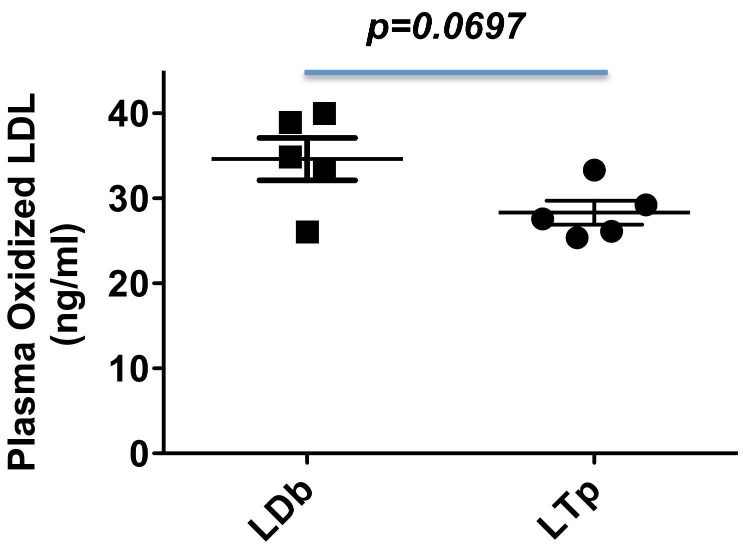

Supplemental Figure S4. Plasma oxidized LDL levels in LDb (Ldlr-/-Apobec1-/-) and LTp (Ldlr-/-Apobec1-/-Pcsk9-/-) . Mouse plasma oxidized LDL (OxLDL) in LDb and LTp mice were determined using ELISA kit from LSBio, Inc., Seattle, WA. The levels of OxLDL in LDb and LTp mice were 35±2.5 and 28±1.4, respectively. The differences were not significant (p=0.0697)

(I)

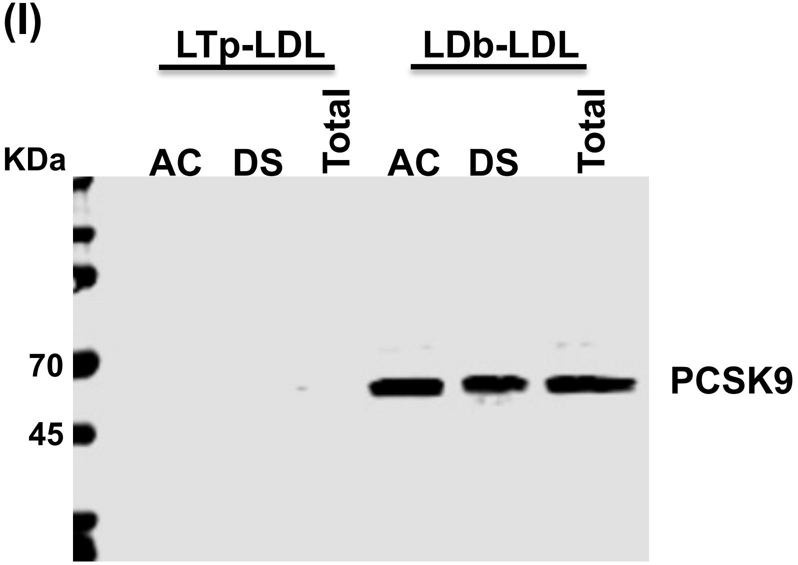

Supplemental Figure S5A: The levels of PCSK9 in Ascending (AC) and Descending (DS) fractions of LDb-LDL. We used FPLC to separate plasma lipoproteins to VLDL, LDL and HDL fractions. In here, we pooled LDL fractions #12-17 of ascending peak of LDL as AC-LDL and LDL fractions of #18-23 descending peak of LDL as DS-LDL. The pool fractions of AC (20 µg) and DS (20 µg), as well as non-fractionated LDL (20 µg) from LDb and LTp mice were separated by SDS-PAGE, followed by Western blot analysis using anti-PCSK9 (Biolegend, 1:3000 dilution). The position of PCSK9 is shown. LTP-LDL has no detectable PCSK9

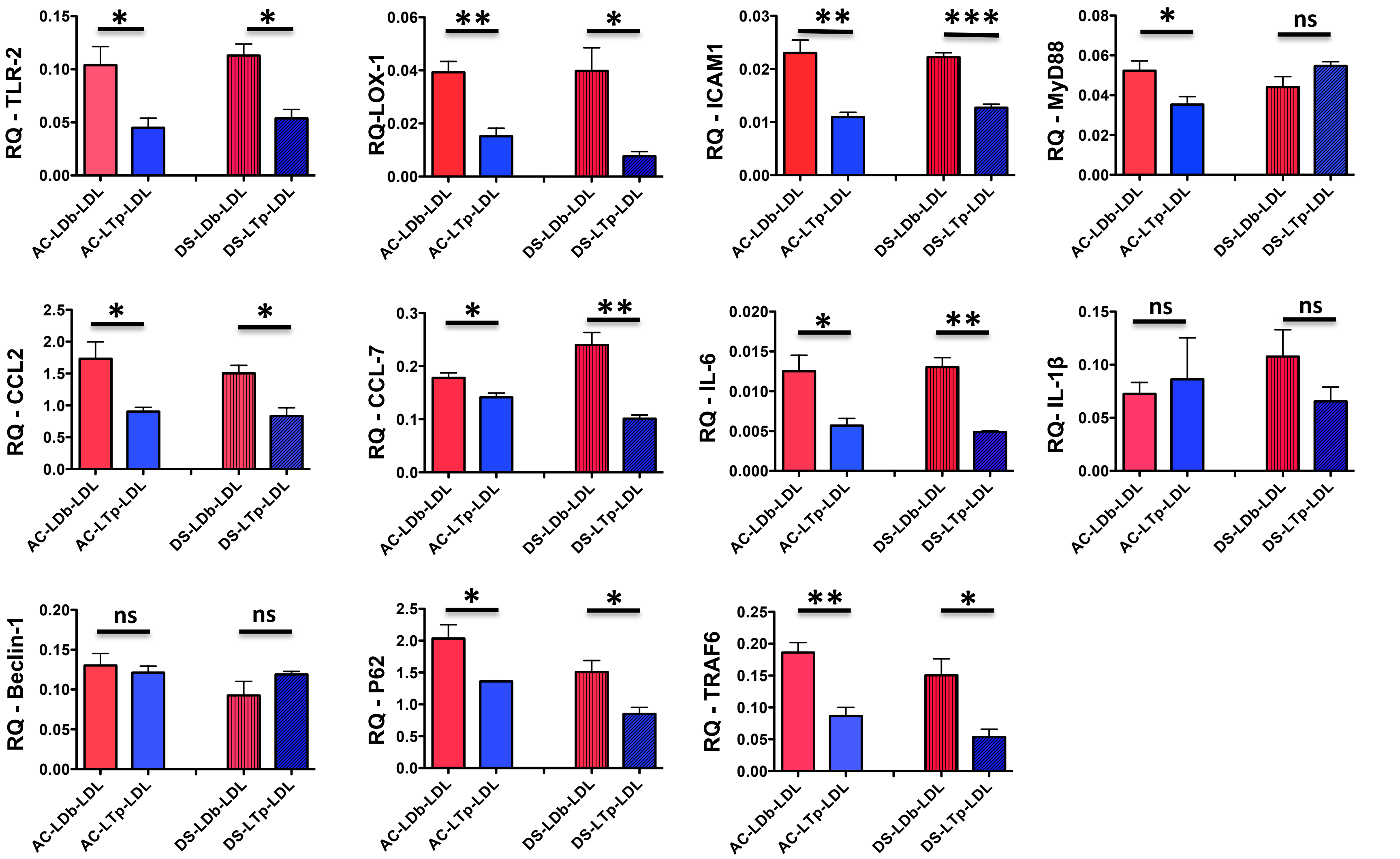

Supplemental Figure S5B. Both the ascending and descending LDb-LDLs stimulated higher gene expressions of pro-atherosclerosis and autophagy molecules than that of LTp-LDL on MCECs. Mouse cardiac endothelial cells (MCECs) were plated onto 12-well plates in duplicate. On the next day each well was incubated with either AC-LDb-LDL (20 μg/ml), or AC-LTp-LDL (20 μg/ml), or DS-LDb-LDL (20 μg/ml), or DS-LTp-LDL (20 μg/ml) as indicated for 24 h. Cells were collected to extract RNA. The experiment was performed three times. The mRNA levels were measured by real-time quantitative RT-PCR and normalized by beta-Actin. The results are expressed as RQ; mean±SEM. Statistical analyses were performed using *two-tailed unpaired t-tests with Welch's correction*. (* $p<0.05$, ** $p<0.005$, *** $p<0.0005$).
